# Development of therapeutic antibodies for the treatment of diseases

**DOI:** 10.1186/s12929-019-0592-z

**Published:** 2020-01-02

**Authors:** Ruei-Min Lu, Yu-Chyi Hwang, I-Ju Liu, Chi-Chiu Lee, Han-Zen Tsai, Hsin-Jung Li, Han-Chung Wu

**Affiliations:** 1 Institute of Cellular and Organismic Biology, Academia Sinica, Taipei, 115 Taiwan; 2 128 Academia Rd., Section 2, Nankang, Taipei, 11529 Taiwan

**Keywords:** Therapeutic antibody, Antibody market, Humanized antibody, Phage display, Human antibody mouse, Single B cell antibody technology, Affinity maturation

## Abstract

It has been more than three decades since the first monoclonal antibody was approved by the United States Food and Drug Administration (US FDA) in 1986, and during this time, antibody engineering has dramatically evolved. Current antibody drugs have increasingly fewer adverse effects due to their high specificity. As a result, therapeutic antibodies have become the predominant class of new drugs developed in recent years. Over the past five years, antibodies have become the best-selling drugs in the pharmaceutical market, and in 2018, eight of the top ten bestselling drugs worldwide were biologics. The global therapeutic monoclonal antibody market was valued at approximately US$115.2 billion in 2018 and is expected to generate revenue of $150 billion by the end of 2019 and $300 billion by 2025. Thus, the market for therapeutic antibody drugs has experienced explosive growth as new drugs have been approved for treating various human diseases, including many cancers, autoimmune, metabolic and infectious diseases. As of December 2019, 79 therapeutic mAbs have been approved by the US FDA, but there is still significant growth potential. This review summarizes the latest market trends and outlines the preeminent antibody engineering technologies used in the development of therapeutic antibody drugs, such as humanization of monoclonal antibodies, phage display, the human antibody mouse, single B cell antibody technology, and affinity maturation. Finally, future applications and perspectives are also discussed.

## Background

Monoclonal antibodies (mAbs) are produced by B cells and specifically target antigens. The hybridoma technique introduced by Köhler and Milstein in 1975 [1] has made it possible to obtain pure mAbs in large amounts, greatly enhancing the basic research and potential for their clinical use. Other scientific and technological advances have also enabled the successful translation of mAbs to the clinic. Around the world, at least 570 therapeutic mAbs have been studied in clinical trials by commercial companies [2], and 79 therapeutic mAbs have been approved by the United States Food and Drug Administration (US FDA) and are currently on the market [3], including 30 mAbs for the treatment of cancer (Table 1).

**Table 1:** US FDA-approved monoclonal antibody on the market

mAb	Brand name	Company	Target	Format	Technology	Indication^&^	US^#^ Approval
Muromonab-CD3	Orthoclone OKT3	Centocor Ortho Biotech Products LP.	CD3	Murine IgG2a	Hybridoma/Janssen Biotech, Inc	Kidney transplant rejection	1986*
Abciximab	Reopro	Centocor Inc./Eli Lilly/Janssen Biotech Inc.	GPIIb/IIIa	Chimeric IgG1 Fab	Hybridoma	Prevention of blood clots in angioplasty	1994
Rituximab	MabThera, Rituxan	Biogen Inc./Roche, F. Hoffmann-La Roche Ltd./Genentech Inc.	CD20	Chimeric IgG1	Hybridoma	Non-Hodgkin lymphoma	1997
Palivizumab	Synagis	MedImmune/AbbVie Inc.	RSV	Humanized IgG1	Hybridoma	Prevention of respiratory syncytial virus infection	1998
Infliximab	Remicade	Janssen Biotech Inc.	TNFα	Chimeric IgG1	Hybridoma	Crohn’s disease	1998
Trastuzumab	Herceptin	Roche, F. Hoffmann-La Roche, Ltd./Genentech Inc.	HER2	Humanized IgG1	Hybridoma	Breast cancer	1998
Alemtuzumab	Campath, Lemtrada	Berlex Inc./Genzyme Corp./Millennium Pharmaceuticals Inc.	CD52	Humanized IgG1	Hybridoma	Chronic myeloid leukemia	2001
Adalimumab	Humira	AbbVie Inc.	TNFα	Human IgG1	Phage display	Rheumatoid arthritis	2002
Ibritumomab tiuxetan	Zevalin	Biogen Inc./Schering AG/Spectrum Pharmaceuticals Inc.	CD20	Murine IgG1	Hybridoma	Non-Hodgkin lymphoma	2002
Omalizumab	Xolair	Roche, F. Hoffmann-La Roche, Ltd./Genentech Inc./Novartis Pharmaceuticals Corp./Tanox Inc.	IgE	Humanized IgG1	Hybridoma	Asthma	2003
Cetuximab	Erbitux	Bristol-Myers Squibb/Merck & Co. Inc./Eli Lilly/ImClone Systems Inc.	EGFR	Chimeric IgG1	Hybridoma	Colorectal cancer	2004
Bevacizumab	Avastin	Roche, F. Hoffmann-La Roche, Ltd./Genentech Inc.	VEGF-A	Humanized IgG1	Hybridoma	Colorectal cancer	2004
Natalizumab	Tysabri	Biogen Inc./Elan Pharmaceuticals International, Ltd.	ITGA4	Humanized IgG4	Hybridoma	Multiple sclerosis	2004
Panitumumab	Vectibix	Amgen	EGFR	Human IgG2	Transgenic mice	Colorectal cancer	2006
Ranibizumab	Lucentis	Roche, F. Hoffmann-La Roche Ltd./Genentech Inc./Novartis Pharmaceuticals Corp.	VEGF-A	Humanized IgG1 Fab	Hybridoma	Macular degeneration	2006
Eculizumab	Soliris	Alexion Pharmaceuticals Inc.	C5	Humanized IgG2/4	Hybridoma	Paroxysmal nocturnal hemoglobinuria	2007
Certolizumab pegol	Cimzia	Celltech, UCB.	TNFα	Humanized Fab, pegylated	Hybridoma	Crohn’s disease	2008
Ustekinumab	Stelara	Medarex/Centocor Ortho Biotech Inc./Janssen Biotech Inc.	IL-12/23	Human IgG1	Transgenic mice	Psoriasis	2009
Canakinumab	Ilaris	Novartis Pharmaceuticals Corp.	IL-1β	Human IgG1	Transgenic mice	Muckle-Wells syndrome	2009
Golimumab	Simponi	Centocor Ortho Biotech Inc./Janssen Biotech Inc.	TNFα	Human IgG1	Transgenic mice	Rheumatoid and psoriatic arthritis, ankylosing spondylitis	2009
Ofatumumab	Arzerra	Genmab A/S /GlaxoSmithKline /Novartis.	CD20	Human IgG1	Transgenic mice	Chronic lymphocytic leukemia	2009
Tocilizumab	RoActemra, Actemra	Chugai Pharmaceutical Co., Ltd./Roche, F. Hoffmann-La Roche. Ltd./Genentech Inc.	IL-6R	Humanized IgG1	Hybridoma	Rheumatoid arthritis	2010
Denosumab	Xgeva, Prolia	Amgen	RANKL	Human IgG2	Transgenic mice	Bone loss	2010
Belimumab	Benlysta	GlaxoSmithKline /Human Genome Sciences Inc.	BLyS	Human IgG1	Phage display	Systemic lupus erythematosus	2011
Ipilimumab	Yervoy	Bristol-Myers Squibb/Medarex	CTLA-4	Human IgG1	Transgenic mice	Metastatic melanoma	2011
Brentuximab vedotin	Adcetris	Seattle genetics Inc./Takeda Pharmaceutical Co., Ltd.	CD30	Chimeric IgG1; ADC	Hybridoma	Hodgkin lymphoma, systemic anaplastic large cell lymphoma	2011
Pertuzumab	Perjeta	Roche, F. Hoffmann-La Roche, Ltd./Genentech Inc.	HER2	Humanized IgG1	Hybridoma	Breast Cancer	2012
Trastuzumab emtansine	Kadcyla	Roche, F. Hoffmann-La Roche Ltd./Genentech Inc./ImmunoGen Inc.	HER2	Humanized IgG1; ADC	Hybridoma	Breast cancer	2012
Raxibacumab	Abthrax	GlaxoSmithKline /Human Genome Sciences Inc. (HGSI)	*B. anthrasis* PA	Human IgG1	Transgenic mice	Anthrax infection	2012
Obinutuzumab	Gazyva, Gazyvaro	Biogen Inc./Roche, F. Hoffmann-La Roche, Ltd./Genentech Inc.	CD20	Humanized IgG1 Glycoengineered	Hybridoma	Chronic lymphocytic leukemia	2013
Siltuximab	Sylvant	Centocor Inc./Janssen Biotech Inc./Janssen-Cilag International NV	IL-6	Chimeric IgG1	Hybridoma	Castleman disease	2014
Ramucirumab	Cyramza	Eli Lilly/ImClone Systems Inc.	VEGFR2	Human IgG1	Phage display	Gastric cancer	2014
Vedolizumab	Entyvio	Genentech Inc./Millennium Pharmaceuticals Inc./Takeda Pharmaceuticals U.S.A. Inc.	α4β7 integrin	Humanized IgG1	Hybridoma	Ulcerative colitis, Crohn disease	2014
Blinatumomab	Blincyto	Amgen	CD19, CD3	Murine bispecific tandem scFv	Hybridoma	Acute lymphoblastic leukemia	2014
Nivolumab	Opdivo	Bristol-Myers Squibb/Ono Pharmaceutical Co., Ltd.	PD-1	Human IgG4	Transgenic mice	Melanoma, non-small cell lung cancer	2014
Pembrolizumab	Keytruda	Merck & Co. Inc.	PD-1	Humanized IgG4	Hybridoma	Melanoma	2014
Idarucizumab	Praxbind	Boehringer Ingelheim Pharmaceuticals	Dabigatran	Humanized Fab	Hybridoma	Reversal of dabigatran-induced anticoagulation	2015
Necitumumab	Portrazza	Eli Lilly/ImClone Systems Inc.	EGFR	Human IgG1	Phage display	Non-small cell lung cancer	2015
Dinutuximab	Unituxin	United Therapeutics Corporation	GD2	Chimeric IgG1	Hybridoma	Neuroblastoma	2015
Secukinumab	Cosentyx	Novartis Pharmaceuticals Corp.	IL-17α	Human IgG1	Transgenic mice	Psoriasis	2015
Mepolizumab	Nucala	Centocor Inc./GlaxoSmithKline	IL-5	Humanized IgG1	Hybridoma	Severe eosinophilic asthma	2015
Alirocumab	Praluent	Regeneron Pharmaceuticals Inc./Sanofi.	PCSK9	Human IgG1	Transgenic mice	High cholesterol	2015
Evolocumab	Repatha	Amgen/Amgen Astellas BioPharma K.K.	PCSK9	Human IgG2	Transgenic mice	High cholesterol	2015
Daratumumab	Darzalex	Genmab A/S/Janssen Biotech Inc.	CD38	Human IgG1	Transgenic mice	Multiple myeloma	2015
Elotuzumab	Empliciti	Bristol-Myers Squibb/AbbVie Inc.	SLAMF7	Humanized IgG1	Hybridoma	Multiple myeloma	2015
Ixekizumab	Taltz	Eli Lilly	IL-17α	Humanized IgG4	Hybridoma	Psoriasis	2016
Reslizumab	Cinqaero, Cinqair	Celltech, UCB/Schering-Plough/Teva Pharmaceutical Industries, Ltd.	IL-5	Humanized IgG4	Hybridoma	Asthma	2016
Olaratumab	Lartruvo	Eli Lilly/ImClone Systems Inc.	PDGFRα	Human IgG1	Transgenic mice	Soft tissue sarcoma	2016
Bezlotoxumab	Zinplava	Merck & Co. Inc.	*Clostridium difficile* enterotoxin B	Human IgG1	Transgenic mice	Prevention of *Clostridium difficile* infection recurrence	2016
Atezolizumab	Tecentriq	Roche, F. Hoffmann-La Roche, Ltd./Genentech Inc.	PD-L1	Humanized IgG1	Hybridoma	Bladder cancer	2016
Obiltoxaximab	Anthim	Elusys Therapeutics Inc.	*B. anthrasis* PA	Chimeric IgG1	Hybridoma	Prevention of inhalational anthrax	2016
Inotuzumab ozogamicin	Besponsa	Wyeth Pharmaceuticals/Pfizer.	CD22	Humanized IgG4	Hybridoma	Acute lymphoblastic leukemia	2017
Brodalumab	Siliq, Lumicef	MedImmune/Amgen/Kyowa Hakko Kirin /AstraZeneca/Valeant Pharmaceuticals International Inc.	IL-17R	Human IgG2	Transgenic mice	Plaque psoriasis	2017
Guselkumab	Tremfya	MorphoSys/Janssen Biotech Inc.	IL-23 p19	Human IgG1	Phage display	Plaque psoriasis	2017
Dupilumab	Dupixent	Regeneron Pharmaceuticals Inc./Sanofi	IL-4Rα	Human IgG4	Transgenic mice	Atopic dermatitis	2017
Sarilumab	Kevzara	Regeneron Pharmaceuticals Inc./Sanofi	IL-6R	Human IgG1	Transgenic mice	Rheumatoid arthritis	2017
Avelumab	Bavencio	Merck Serono International S.A./Pfizer	PD-L1	Human IgG1	Phage display	Merkel cell carcinoma	2017
Ocrelizumab	Ocrevus	Biogen Inc./Roche, F. Hoffmann-La Roche, Ltd./Genentech Inc./SIGMA-TAU Industrie Farmaceutiche Riunite S.p.A.	CD20	Humanized IgG1	Hybridoma	Multiple sclerosis	2017
Emicizumab	Hemlibra	Chugai Pharmaceutical Co., Ltd./Roche, F. Hoffmann-La Roche, Ltd.	Factor IXa, X	Humanized IgG4, bispecific	Hybridoma	Hemophilia A	2017
Benralizumab	Fasenra	MedImmune/Kyowa Hakko Kirin/AstraZeneca	IL-5Rα	Humanized IgG1	Hybridoma	Asthma	2017
Gemtuzumab ozogamicin	Mylotarg	Pfizer	CD33	Humanized IgG4; ADC	Hybridoma	Acute myeloid leukemia	2017
Durvalumab	Imfinzi	MedImmune/AstraZeneca	PD-L1	Human IgG1	Transgenic mice	Bladder cancer	2017
Burosumab	Crysvita	Kyowa Hakko Kirin/Ultragenyx Pharmaceutical Inc.	FGF23	Human IgG1	Transgenic mice	X-linked hypophosphatemia	2018
Lanadelumab	Takhzyro	Dyax Corp.	Plasma kallikrein	Human IgG1	Phage display	Hereditary angioedema attacks	2018
Mogamulizumab	Poteligeo	Kyowa Hakko Kirin	CCR4	Humanized IgG1	Hybridoma	Mycosis fungoides or Sézary syndrome	2018
Erenumab	Aimovig	Novartis	CGRPR	Human IgG2	Transgenic mice	Migraine prevention	2018
Galcanezumab	Emgality	Eli Lilly	CGRP	Humanized IgG4	Hybridoma	Migraine prevention	2018
Tildrakizumab	Ilumya	Merck & Co. Inc./Sun Pharmaceutical Industries, Ltd.	IL-23 p19	Humanized IgG1	Hybridoma	Plaque psoriasis	2018
Cemiplimab	Libtayo	Regeneron Pharmaceuticals Inc.	PD-1	Human mAb	Transgenic mice	Cutaneous squamous cell carcinoma	2018
Emapalumab	Gamifant	NovImmmune	IFNγ	Human IgG1	Phage display	Primary hemophagocytic lymphohistiocytosis	2018
Fremanezumab	Ajovy	Teva Pharmaceutical Industries, Ltd.	CGRP	Humanized IgG2	Hybridoma	Migraine prevention	2018
Ibalizumab	Trogarzo	Taimed Biologics Inc./Theratechnologies Inc.	CD4	Humanized IgG4	Hybridoma	HIV infection	2018
Moxetumomab pasudodox	Lumoxiti	MedImmune/AstraZeneca	CD22	Murine IgG1 dsFv	Phage display	Hairy cell leukemia	2018
Ravulizumab	Ultomiris	Alexion Pharmaceuticals Inc.	C5	humanized IgG2/4	Hybridoma	Paroxysmal nocturnal hemoglobinuria	2018
Caplacizumab	Cablivi	Ablynx	von Willebrand factor	Humanized Nanobody	Hybridoma	Acquired thrombotic thrombocytopenic purpura	2019
Romosozumab	Evenity	Amgen/UCB	Sclerostin	Humanized IgG2	Hybridoma	Osteoporosis in postmenopausal women at increased risk of fracture	2019
Risankizumab	Skyrizi	Boehringer Ingelheim Pharmaceuticals/ AbbVie Inc.	IL-23 p19	Humanized IgG1	Hybridoma	Plaque psoriasis	2019
Polatuzumab vedotin	Polivy	Roche, F. Hoffmann-La Roche, Ltd.	CD79β	Humanized IgG1 ADC	Hybridoma	Diffuse large B-cell lymphoma	2019
Brolucizumab	Beovu	Novartis Pharmaceuticals Corp.	VEGF-A	Humanized scFv	Hybridoma^$^	Macular degeneration	2019
Crizanlizumab	Adakveo	Novartis Pharmaceuticals Corp.	P-selectin	Humanized IgG2	Hybridoma	Sickle cell disease	2019

*Marketing end date on July 30th, 2011

^#^Year of the first US FDA approval

^&^Indication of the first US FDA approval

^$^Rabbit hybridoma technology

**Fig. 1:**
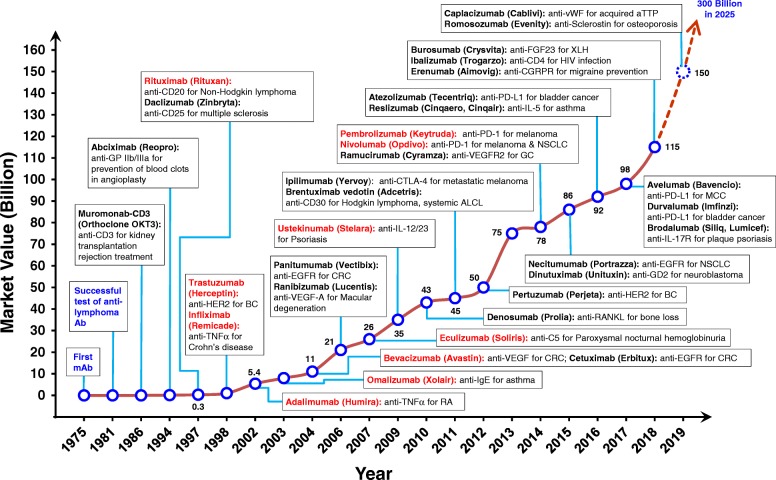
Timeline from 1975 showing the successful development of therapeutic antibodies and their applications. Many biotech companies that promised antibodies as anticancer “magic bullets” were launched from 1981 to 1986. The height of the line and numerical annotations represent the estimated market value of mAb therapeutics in each indicated year (shown as billions of US dollars). Antibodies colored in red represent the top 10 best-selling antibody drugs in 2018. Ab, antibody; ALCL, systematic anaplastic large-cell lymphoma; aTTP, acquired thrombotic thrombocytopenic purpura; BC, breast cancer; CD, cluster of differentiation; CGRP, calcitonin gene-related peptide; CGRPR, calcitonin gene-related peptide receptor; CRC, colorectal cancer; CTLA-4, cytotoxic T-lymphocyte-associated protein 4; EGFR, epidermal growth factor receptor; FGF, fibroblast growth factor; GC, gastric cancer; GD2, disialoganglioside G_D2_; HER2, human epidermal growth factor receptor 2; IgE, immunoglobulin E; IL, interleukin; IL-17R, interleukin-17 receptor; mAb, monoclonal antibody; MCC, merkel-cell carcinoma; NSCLC, non-small cell lung cancer; PD-1, programmed cell death protein 1; PD-L1, programmed death-ligand 1; TNFα, tumor necrosis factor α; RA, rheumatoid arthritis; RANKL, receptor activator of nuclear factor kappa-B ligand; VEGF-A, vascular endothelial growth factor A; VEGFR2, vascular endothelial growth factor receptor 2; vWF, von Willebrand factor; XLH, X-linked hypophosphatemia

The increasing importance of therapeutic mAbs is apparent (Fig. 1), as mAbs have become the predominant treatment modality for various diseases over the past 25 years. During this time, major technological advances have made the discovery and development of mAb therapies quicker and more efficient. Since 2008, 48 new mAbs have been approved, contributing to a total global market of 61 mAbs in clinical use at the end of 2017, according to the US FDA. Strikingly, a total of 18 new antibodies were granted approval by the US FDA from 2018 to 2019 – this number was tallied from information contained on various websites, including the antibody society [3], the database of therapeutic antibodies [4], and company pipelines and press releases. A list of antibody-based drugs approved by the US FDA is shown in Table 1.

**Fig. 2:**
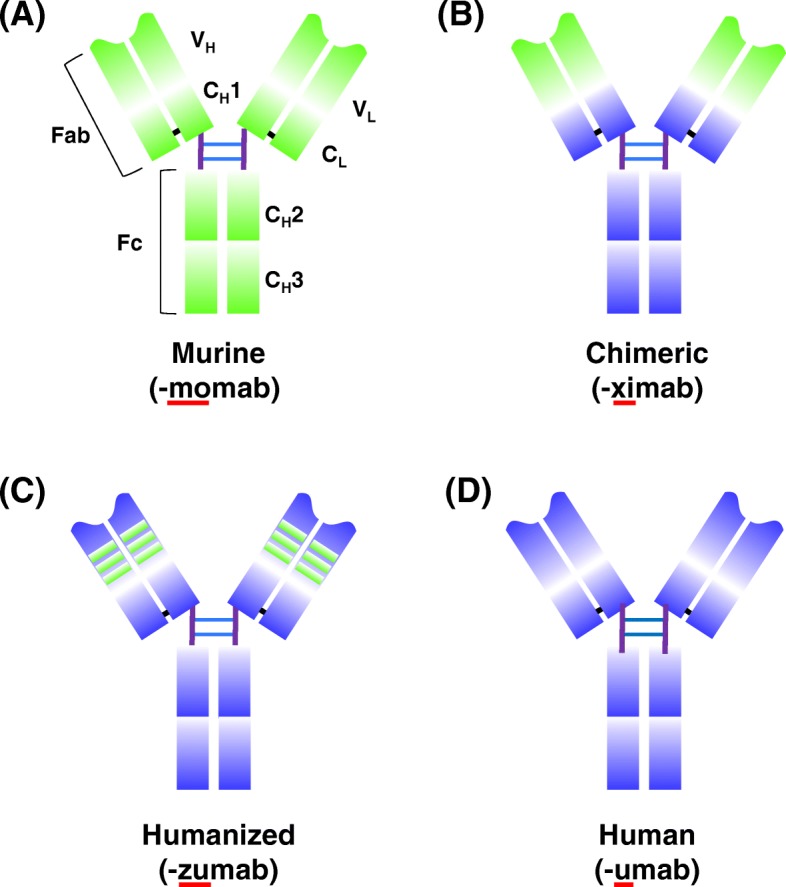
Schematic overview of antibody humanization from murine antibodies (green domains) to fully human antibodies (orange domains) and associated suffixes. **a** The murine monoclonal antibody. **b** The chimeric monoclonal antibody: variable regions are of murine origin, and the rest of the chains are of human origin. **c** Humanized monoclonal antibody: only includes the hypervariable segments of murine origin. **d** Human monoclonal. C_H_: domains of the constant region of the heavy chain; C_L_: constant domain of the light chain; Fab and Fc: fragments resulting from proteolysis; V_H_: variable domain of the heavy chain; V_L_: variable domain of the light chain

The first therapeutic mAb, muromonab-CD3 (Orthoclone OKT3), was approved by the US FDA in 1986 [5] and comprises a murine mAb against T cell-expressed CD3 that functions as an immunosuppressant for the treatment of acute transplant rejection. The marketing end date of muromonab-CD3 is on July 30th, 2011 (Table 1). To overcome problems of decreased immunogenic potential and efficacy, while making possible the therapeutic use of antibodies for an extended duration, researchers developed techniques to transform rodent antibodies into structures more similar to human antibodies, without loss of binding properties. The first chimeric antibody, anti-GPIIb/IIIa antigen-binding fragment (Fab) (abciximab), was approved in 1994 by the US FDA for inhibition of platelet aggregation in cardiovascular diseases (Fig. 1). The drug was developed by combining sequences of the murine variable domain with human constant region domain (Fig. 2b) [6, 7]. Then the first mAb with an oncologic indication, rituximab, a chimeric anti-CD20 IgG1 approved for non-Hodgkin’s lymphoma in 1997 by US FDA (Fig. 1) [8, 9].

One exceptional advance that accelerated the approval of therapeutic mAbs was the generation of humanized antibodies by the complementary-determining region (CDR) grafting technique [10]. In CDR grafting, non-human antibody CDR sequences are transplanted into a human framework sequence in order to maintain target specificity [10] (Fig. 2c). The first humanized mAb approved by the US FDA in 1997 was the anti-IL-2 receptor, daclizumab, for the prevention of transplant rejection (Fig. 1) [11]. The humanization of antibodies made it possible to clinically apply a new class of biologics directed against diseases that require long-term treatment, such as cancer and autoimmune diseases [12].

**Fig. 3:**
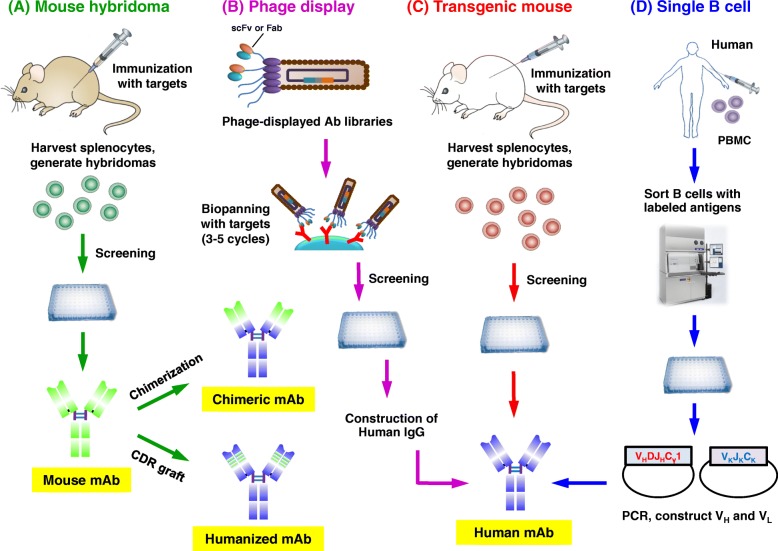
Approaches for the development of therapeutic antibodies. **a** The traditional mouse hybridoma technique starts by immunization of mice with desired antigens to trigger an immune response. Harvested splenocytes are fused with myeloma cells to produce hybridoma cells that persistently secrete antibodies. After the screening, selected leads are used to generate chimeric or humanized antibodies. **b** Phage display. A human phage-displayed human antibody library is used to select antigens of interest. After 3–5 rounds of biopanning, immuno-positive phage clones are screened by ELISA; then DNA sequences are analyzed to construct and express human IgGs. **c** Transgenic mouse. Similar to the mouse hybridoma technique or single B cell methods. **d** The single B cell technique. From infected or vaccinated donors, PBMCs are prepared for isolation of suitable B cells by flow cytometry. Following the RT-PCR, V_H_ and V_L_ information of each B cell informs the generation of human mAbs

Based on the success of humanized mAbs in the clinic, a key discovery technology to obtain fully human mAbs (Fig. 2d) was developed in 1990 by Sir Gregory P. Winter [10, 13]. This technique was based on phage display, wherein diverse exogenous genes are incorporated into filamentous bacteriophages to compose a library. The library proteins are then presented on the phage surface as fusions with a phage coat protein, allowing the selection of specific binders and affinity characteristics. The phage display technique was first introduced by George P. Smith [14] and comprises a powerful method for the rapid identification of peptides or antibody fragments, such as single chain fragment variable (scFv) or Fab, that bind a variety of target molecules (proteins, cell-surface glycans and receptors) [15] (Fig. 3b). The Nobel Prize in Chemistry 2018 was awarded to George P. Smith and Sir Gregory P. Winter. George Smith developed phage-displayed peptides, which can be used to evolve new proteins [14]. Gregory P. Winter was able to apply the phage-displayed antibody library to the discovery and isolation of antibodies [13]. Phage display technology has also been used for antibody maturation by site-directed mutagenesis of CDR and affinity selection. Based on these techniques, the first fully human therapeutic antibody, adalimumab (Humira), an anti-tumor necrosis factor α (TNFα) human antibody [16], was approved in 2002 by the US FDA for rheumatoid arthritis (Fig. 1). Until now, nine human antibody drugs generated by phage display have been approved by the US FDA (Table 5).

Transgenic animals represent another technology for obtaining fully human mAbs (Fig. 3c). This technology was introduced in 1994 by the publication of two transgenic mouse lines, the HuMabMouse [35] and the XenoMouse [36]. The lines were genetically modified such that human immunoglobulin (Ig) genes were inserted into the genome, replacing the endogenous Ig genes and making these animals capable of synthesizing fully human antibodies upon immunization [35, 37]. The first human antibody generated in a transgenic mouse to anti-epidermal growth factor receptor (EGFR), panitumumab, was approved by the US FDA in 2006 (Fig. 1) [38, 39]. The number of fully human antibodies made from transgenic mice has increased rapidly, with the number of approved drugs currently at 19 (Table 5). Depending on the immunization protocol, high-affinity human antibodies can be obtained through further selection of hybridoma clones generated from immunized transgenic mice. Using a theoretically similar approach, the generation of neutralizing human antibodies from human B cells has also yielded promising results for infectious disease therapeutics.

The recent development of bispecific antibodies offers attractive new opportunities for the design of novel protein therapeutics. A bispecific antibody can be generated by utilizing protein engineering techniques to link two antigen binding domains (such as Fabs or scFvs), allowing a single antibody to simultaneously bind different antigens. Thus, bispecific antibodies may be engineered to exhibit novel functions, which do not exist in mixtures of the two parental antibodies. Most bispecific antibodies are designed to recruit cytotoxic effector cells of the immune system to target pathogenic cells [40]. The first approved bispecific antibody was catumaxomab in Europe in 2009 [41]. Catumaxomab targets CD3 and EpCAM to treat solid tumors in patients with malignant ascites. However, this drug was withdrawn from the market in 2017 for commercial reasons. Currently, two bispecific antibodies have obtained US FDA approval and are on the market. First, blinatumomab is a bispecific T-cell engager (BiTE) that targets CD3 and CD19 for treatment of B-cell precursor acute lymphoblastic leukemia (ALL) [42]. Second, emicizumab is a full-size bispecific IgG with natural architecture, which binds to activated coagulation factors IX and X for the treatment of haemophilia A [43]. To date, there are more than 85 bispecific antibodies in clinical trials, about 86% of which are under evaluation as cancer therapies [40]. The concepts and platforms driving the development of bispecific antibodies continue to advance rapidly, creating many new opportunities to make major therapeutic breakthroughs.

While mAbs are routinely used in biochemistry, molecular and cellular biology, and medical research, perhaps the most beneficial application is their use as therapeutic drugs for the treatment of human diseases, such as cancer, asthma, arthritis, psoriasis, Crohn’s disease, transplant rejection, migraine headaches and infectious diseases (Table 1). Important advances in antibody engineering made over the past decade have enhanced the safety and efficacy of the therapeutic antibodies. These developments, along with a greater understanding of the immunomodulatory properties of antibodies, have paved the way for the next generation of new and improved antibody-based drugs for the treatment of human diseases.

## Clinical applications and market for therapeutic antibodies

### Therapeutic antibodies currently approved as disease treatments

The mAb market enjoys a healthy pipeline and is expected to grow at an increasing pace, with a current valuation of $115.2 billion in 2018 [44]. Despite this high growth potential, new companies are unlikely to take over large shares of the market, which is currently dominated by seven companies: Genentech (30.8%), Abbvie (20.0%), Johnson & Johnson (13.6%), Bristol-Myers Squibb (6.5%), Merck Sharp & Dohme (5.6%), Novartis (5.5%), Amgen (4.9%), with other companies comprising the remaining 13% [44].

Many mAbs products achieved annual sales of over US$3 billion in 2018 (Fig. 1), while six (adalimumab, nivolumab, pembrolizumab, trastuzumab, bevacizumab, rituximab) had sales of more than $6 billion (Table 2). Adalimumab (Humira) had the highest sales figure ever recorded for a biopharmaceutical product, nearly $19.9 billion. The top ten selling mAb products in 2018 are listed in Table 2. Top-selling mAb drugs were ranked based on sales or revenue reported by biological or pharmacological companies in press announcements, conference calls, annual reports or investor materials throughout 2018. For each drug, the name, sponsors, disease indications, and 2018 sales are shown.

**Table 2:** Top 10 best-selling monoclonal antibody drugs in 2018

No.	Drug	Indication (1st US FDA Approval Year)	Company	2018 Revenue (USD)
1	Adalimumab (Humira)	Rheumatoid arthritis (2002) Psoriatic arthritis (2005) Ankylosing spondylitis (2006) Juvenile Idiopathic Arthitis (2008) Psoriasis (2008) Crohn’s disease (2010) Ulcerative colitis (2012) Hidradenitis suppurativa (2015) Uveitis (2018)	AbbVie	$19.9 bn
2	Nivolumab (Opdivo)	Melanoma (2015) Non-small cell lung cancer (2015) Renal cell carcinoma (2015) Head and neck squamous cell (2016)	Bristol-Myers Squibb	$7.6 bn
3	Pembrolizumab (Keytruda)	Melanoma (2014) Head and neck cancer (2016) Non-small cell lung caccer (2015) Lymphoma (2018) Cervical cancer (2018) Microsatellite instability-high cancer (2018)	Merck & Co	$7.2 bn
4	Trastuzumab (Herceptin)	Breast cancer (1998) Gastric cancer (2010)	Roche	$7.0 bn
5	Bevacizumab (Avastin)	Colorectal cancer (2004) Non-small cell lung caccer (2006) Breast ERB2 negative cancer (2008) Renal cell carcinoma (2009) Glioblastoma (2011)	Roche	$6.8 bn
6	Rituximab, (Rituxan)	Non-Hodgkin’s lymphoma (1997) Chronic lymphocytic leukemia (2010) Rheumatoid arthritis (2006) Pemphigus vulgaris (2018)	Roche	$6.8 bn
7	Infliximab (Remicade)	Crohn’s Disease (1998) Rheumatoid arthritis (1999) Ankylosing spondylitis (2004) Ulcerative colitis (2005) Psoriatic arthritis (2005) Psoriasis (2006)	Johnson & Johnson	$5.9 bn
8	Ustekinumab (Stelara)	Psoriasis (2009) Psoriatic arthritis (2013) Crohn’s Disease (2016)	Johnson & Johnson	$5.2 bn
9	Eculizumab (Soliris)	Paroxysmal nocturnal hemoglobinuria (2007) Atypical hemolytic uremic syndrome (2011) Generalized myasthenia gravis (2017) Neuromyelitis optica spectrum disorder (2019)	Alexion	$3.6 bn
10	Omalizumab (Xolair)	Asthma (2003) Chronic idiopathic urticaria (2014)	Roche	$3.0 bn

bn, billion

mAbs are increasingly used for a broad range of targets; oncology, immunology, and hematology remain the most prevalent medical applications [45]. Most mAbs have multiple disease indications and at least one that is cancer-related (lymphoma, myeloma, melanoma, glioblastoma, neuroblastoma, sarcoma, colorectal, lung, breast, ovarian, head and neck cancers). As such, oncological diseases are the medical specialty most accessible to mAb treatments [45]. Moreover, the number of target proteins known to function as either stimulatory or inhibitory checkpoints of the immune system has dramatically expanded, and numerous antibody therapeutics targeting programmed cell death protein 1 (PD-1, cemiplimab, nivolumab, pembrolizumab), its ligand programmed death-ligand 1 (PD-L1, durvalumab, avelumab, atezolizumab) or cytotoxic T-lymphocyte–associated antigen 4 (CTLA-4, ipilimumab) have been granted marketing approvals [46].

Adalimumab (Humira) was the world’s best-selling drug in 2018. Adalimumab is a subcutaneously administered biological disease modifier used for the treatment of rheumatoid arthritis and other TNFα-mediated chronic debilitating diseases. It was originally launched by Abbvie in the United States after gaining approval from the US FDA in 2002. It has been shown that Adalimumab reduces the signs and symptoms of moderate to severe rheumatoid arthritis in adults, and it is also used to treat psoriatic arthritis, ankylosing spondylitis, Crohn's disease, ulcerative colitis, psoriasis, hidradenitis suppurativa, uveitis, and juvenile idiopathic arthritis [47, 48]. It may be used alone or in combination with disease-modifying anti-rheumatic drugs [49].

Immune checkpoints are important for maintaining self-tolerance and tempering physiologic immune responses in peripheral tissues. Therefore, the molecules underlying checkpoints have recently drawn considerable interest in cancer immunotherapy [50]. Both nivolumab (Opdivo) and pembrolizumab (Keytruda) are anti-PD-1 mAbs and were the second and third best-selling mAb drugs in 2018 (Table 2). Nivolumab is a human antibody, which blocks a signal that normally prevents activated T cells from attacking cancer cells. The target for nivolumab is the PD-1 receptor, and the antibody blocks the interaction of PD-1 with its ligands, PD-L1 and PD-L2, releasing PD-1 pathway-mediated immune inhibition [51, 52]. Pembrolizumab is a humanized antibody used in cancer immunotherapy to treat melanoma, lung cancer, head and neck cancer, Hodgkin’s lymphoma, and stomach cancer [53–55]. Pembrolizumab is a first-line treatment for NSCLC if cancer cells overexpresse PD-L1 and have no mutations in EGFR or in anaplastic lymphoma kinase [56, 57]. Large randomized clinical trials indicated that NSCLC patients treated with nivolumab and pembrolizumab (both approved by the US FDA in 2014) showed increased overall survival compared with docetaxel, the standard second-line treatment [58].

A total of 12 new mAbs were approved in the US during 2018. The majority of these products were approved for non-cancer indications, perhaps reflecting the higher approval success rate for antibodies as treatments for other diseases. Three antibodies (erenumab, galcanezumab, and fremaezumab) were approved for migraine prevention, and one (Ibalizumab) is used for human immunodeficiency virus (HIV) infection. The three migraine-preventing drugs, Erenumab (Aimovig), galcanezumab (Emgality), and fremaezumab (Ajovy), are mAbs that block the activity of calcitonin gene-related peptide (CGRP) receptor in migraine etiology [59]. CGRP acts through a heteromeric receptor, which is composed of a G protein-coupled receptor(calcitonin receptor-like receptor: CALCRL) and receptor activity-modifying protein 1 (RAMP1) [60, 61]. Both galcanezumab and fremaezumab bind to CGRP and block its binding to the receptor. However, erenumab is the only one of the three antibodies to target the extracellular domains of human G protein-coupled receptors CALCRL and RAMP1,interfering with the CGRP binding pocket [62].

Many mAbs are under development for treatment of infectious diseases, currently only four have been approved by the US FDA: raxibacumab and obiltoxaximab for treatment of inhalational anthrax [63], palivizumab for prevention of respiratory syncytial virus in high-risk infants [64], and ibalizumab for treatment of HIV infection patients [65]. Ibalizumab (Trogarzo) is a humanized IgG4 mAb that is used as a CD4 domain 2-directed post-attachment HIV-1 inhibitor. The US FDA approved ibalizumab for adult patients infected with HIV who were previously treated and are resistant to currently available therapies.

### Therapeutic antibodies currently in clinical trials

Companies are currently sponsoring clinical studies for more than 570 mAbs. Of these, approximately 90% are early-stage studies designed to assess safety (Phase I) or safety and preliminary efficacy (Phase I/II or Phase II) in patient populations. Most of the mAbs in Phase I (~ 70%) are for cancer treatment, and the proportions of mAbs intended to treat cancer are similar for those currently in Phase II and late-stage clinical studies (pivotal Phase II, Phase II/III or Phase III) [2].

Twenty-nine novel antibody therapeutics were in late-stage clinical studies for non-cancer indications in 2018. Among the trials for these mAbs, no single therapeutic area predominated, but 40% were for immune-mediated disorders, which comprised the largest group. From this group of potential treatments, leronlimab and brolucizumab entered regulatory review by the end of 2018, and five mAbs (eptinezumab, teprotumumab, crizanlizumab, satralizumab, and tanezumab) may enter regulatory review in 2019. In comparison, there were 33 novel antibody therapeutics in late-stage clinical studies for cancer indications in 2018. Antibody therapeutics for solid tumors clearly predominated, with less than 20% of the candidates intended solely for hematological malignancies. Five mAbs (isatuximab, spartalizumab, tafasitamab, dostarlimab, and ublituximab) license applications were submitted to the US FDA in 2019 [2].

Isatuximab is an anti-CD38 IgG1 chimeric mAb under evaluation as a treatment for patients with multiple myeloma (MM). Combinations of isatuximab and different chemotherapies are being tested in three Phase III studies (ICARIA, IKEMA, and IMROZ) on MM patients. The ICARIA study (NCT02990338) is evaluating the effects of isatuximab in combination with pomalidomide and dexamethasone compared to chemotherapy only in patients with refractory or relapsed MM. Pivotal Phase III ICARIA-MM trial results demonstrated that isatuximab combination therapy showed statistically significant improvements compared to pomalidomide and dexamethasone alone in patients with relapsed or refractory MM in 2019. The US FDA has accepted for review the biologics license application for isatuximab for the treatment relapsed or refractory MM patients. The target action date for the FDA decision is April 2020 [66]. The IKEMA (NCT03275285) and IMROZ (NCT03319667) studies are evaluating the isatuximab with other chemotherapeautic combinations in MM patients [67].

Spartalizumab is a humanized IgG4 mAb that binds PD-1 with sub-nanomolar affinity and blocks its interaction with PD-L1/PD-L2, preventing PD-1-mediated inhibitory signaling and leading to T-cell activation. Clinical study of Spartalizumab is underway with a randomized, double-blind, placebo-controlled Phase III COMBI-i study (NCT02967692), which is evaluating the safety and efficacy of dabrafenib and trametinib in combination with spartalizumab compared to matching placebo in previously untreated patients with BRAF V600-mutant unresectable or metastatic melanoma. The primary endpoints of the study are an assessment of dose-limiting toxicities, changes in PD-L1 levels and CD8+ cells in the tumor microenvironment, and progression-free survival. Key secondary endpoints are overall survival, overall response rate and duration of response. The estimated primary completion date of the study is September 2019 [68].

Dostarlimab is an anti-PD-1 mAb that may be useful as a treatment for several types of cancers. GlaxoSmithKline announced results from a Phase I dose escalation and cohort expansion study (GARNET; NCT02715284) in 2018, which is expected to support a biologics license application submission to the US FDA in 2019. Dostarlimab is being assessed in patients with advanced solid tumors who have limited available treatment options in the GARNET study. The drug is administered at a dose of 500 mg every 3 weeks for the first 4 cycles, and 1000 mg every 6 weeks thereafter in four patient cohorts: microsatellite instability high (MSI-H) endometrial cancer, MSI-H non-endometrial cancer, microsatellite-stable endometrial cancer, and non-small cell lung cancer. Dostarlimab is also being evaluated in another Phase III study (NCT03602859), which is comparing platinum-based therapy with dostarlimab and niraparib versus standard of care platinum-based therapy as first-line treatment of Stage III or IV non-mucinous epithelial ovarian cancer [69].

Ublituximab is a glyco-engineered anti-CD20 antibody currently under clinical investigation in five late-stage clinical studies for different cancers (chronic lymphocytic leukemia, CLL, non-Hodgkin’s lymphoma) and non-cancer (multiple sclerosis) indications. Three Phase III studies are exploring the efficacy of ublituximab in combination with other anti-cancer agents. Among these studies, the UNITY-CLL Phase III study (NCT02612311) is evaluating the combination of ublituximab and TGR-1202, a PI3K delta inhibitor, compared to anti-CD20 obinutuzumab plus chlorambucil in untreated and previously treated CLL patients. Two other Phase III studies (ULTIMATE 1, NCT03277261 and ULTIMATE 2, NCT03277248) are evaluating the efficacy and safety of ublituximab compared to teriflunomide in 440 patients with relapsing multiple sclerosis [70].

## Methodologies for developing therapeutic antibodies

Human, humanized, chimeric, and murine antibodies respectively account for 51, 34.7, 12.5, and 2.8% of all mAbs in clinical use, making human and humanized mAbs the dominant modalities in the field of therapeutic antibodies. In the next section, we first introduce techniques for antibody humanization. Then, we describe three technical platforms related to the generation of fully human antibodies, including phage display, transgenic mice and single B cell antibody isolation (Fig. 3). Last, we describe the use of an affinity maturation method to optimize antibody binding activity.

### Humanization of mAbs

Due to the availability, low cost and quick production time for mouse mAbs, humanization of mouse mAbs has been implemented on a large scale. Non-humanized murine mAbs have many disadvantages as treatments. For example, patients treated with mouse mAbs will produce a rapid human anti-mouse antibody (HAMA) response. HAMAs will not only hasten the clearance of mouse mAbs but may also produce undesirable allergic reactions and tumor penetration. Moreover, the ability of patients to initiate antibody-dependent cellular cytotoxicity (ADCC) in response to murine fragment crystallizable region (Fc) is limited. On the other hand, humanized mAbs are able to effectively exert effector functions while decreasing the immunogenicity of murine antibodies.

#### Generation of humanized mAbs

Humanized mAbs, of which only the CDRs of the light and heavy chains are murine, entered clinical development for the first time in 1988 [71, 72]. CDR grafting is one of the most popular techniques in the production of humanized mAbs and was originally developed by Gregory P. Winter in 1986 [9]. Using this technology, non-human CDR sequences are transplanted into human framework sequences, allowing the antibody to maintain the binding activity to the target antigen [9]. The first US FDA approved CDR-grafted humanized mAb occurred in 1997 for daclizumab, which binds the IL-2 receptor and is used to prevent transplant rejection [11]. Queen and collaborators [73] developed daclizumab not only using CDR grafting, but also using the human framework that is maximally homologous to the murine framework, in order to decrease the loss of antigen recognition. In some cases, certain amino acids in the murine framework are crucial to maintain antibody binding activity. These residues may cooperate with CDRs to present an antibody paratope or directly interact with antigens. Currently, these crucial framework residues can be identified by observing the structure of antibody-antigen complex by X-ray crystallography, cryo-electron microscopy and computer-aided protein homology modelling [74]. The positions of amino acids in the framework may then be considered for restore by ‘human back to mouse’ mutations in CDR-grafted humanized antibodies, thereby improving the affinity and stability of the final product. Currently, web servers are being developed by integrated bioinformatics and antibody structure databases for rendering humanization experiments [75, 76]. They provide the tools for human template selection, grafting, back-mutation evaluation, and antibody modeling. However, if the binding activity of antibodies is still compromised, it should be further performed affinity maturation to improve this situation.

Multiple methods have been developed to quantify the humanness of the variable region of mAbs. Abhinandan and Martin designed a tool called “H-score” to assess the “degree of humanness” of antibody sequences, which calculates the mean sequence identity compared to a subset of human variable region sequences database [77]. A germinality index was defined subsequent to assist germline humanization of a macaque antibody [78]. G-score was derived from the H-score to improve classification of germline framework sequence [79]. T20 score analyzer was established under a large database of ~ 38,700 human antibody variable region sequences to clearly separate human sequences from mouse sequences and many other species as well [80]. It was used to reveal similarities between humanized antibodies and fully human antibodies. These humanness score tools are available online and allow assisting the generation of humanized antibody [80].

The use of humanized antibodies has helped greatly to improve clinical tolerance of mAb therapeutics. Such intricate control over antibody sequences has opened the door to engineering mAbs for a wide range of possible applications in medicine. Currently, half of all mAbs used to treat humans are chimeric or humanized (Fig. 2, Table 1). One of the most well-known humanized antibodies is Trastuzumab (Herceptin), which was approved in 1998 and achieved annual sales of over $7 billion in 2018 (Table 2). Trastuzumab is used for the treatment of patients with human epidermal growth factor receptor 2 (HER2)-positive metastatic breast cancer and gastroesophageal junction adenocarcinoma [57, 58].

#### Immunogenicity of antibody-based therapeutics

The use of mAbs in a clinical setting should have several essential biophysical properties, including high antigen binding activity, high stability, and low immunogenicity [81]. Antibody immunogenicity means the degree of the host immune system can recognize and react to these therapeutic agents. Anti-drug antibodies (ADA) induced by the immune system can be found while immunogenicity occurring in patients administered with antibody drugs. Anti-drug antibodies have the potential to neutralize therapeutic agents, which can reduce the efficacy of the drugs [82]. Importantly, anti-drug antibodies may further cause adverse effects ranging from skin rashes to systemic inflammatory responses in the patients, which can impact both safety and efficacy of the antibody drugs in clinic use [83]. Immunogenicity is influenced by several factors, such as drug dosage, administration strategy (route and combination), impurities contamination, aggregates arising from Ab/Ag binding complex, and structural features (sequence variation and glycosylation) [84].

Humanized antibodies harbor human sequence in constant regions and nearly all human sequence in Fv, of which only CDRs are murine grafted. Antibodies of more human-like usually allow them to be higher tolerant and lower immunogenic in a clinical setting. For example, Perpetua et al. showed a case to support this concept [85]. They compared a humanized anti-CD52 antibody with its parental murine version and demonstrated humanization offers a significant reduction in immunogenicity. However, humanized antibodies retain murine CDRs which could be regarded as foreign antigens by host immune systems and eventually arise immunogenicity. For example, ADA was detected in 0.5% of women with metastatic breast cancer, who were treated with Trastuzumab during their therapeutic courses [86]. Recently, an immunogenicity analysis result from clinical data showed the ADA rates were 7.1% (21/296) in the HER-2 positive breast cancer patients with treatment of Trastuzumab [87]. The variation of immunogenicity in the same antibody drug may be caused by many potential factors: the age, race, genetic background, other related diseases, and programs of drugs administration.

The CDRs and frameworks of fully human antibodies are derived for human immunoglobulin gene repertoires, thus which can theoretically bypass immunogenicity. However, several fully human antibodies have been reported to induce marked immune responses when administrated in patients [88]. Adalimumab (Humira), a human IgG1, has been reported to generate significant immune responses through eliciting anti-idiotypic antibody in a part of patients (5–89%) which varies depending on the disease and the therapy [89, 90]. Golimumab (Simponi), a fully human anti-TNFα antibody, combining with methotrexate for treatment of rheumatoid arthritis cause 16% of patients producing anti-drug antibodies [91]. One reason of these scenarios is that Fv sequence of human antibodies is not identical to human germline: antibody evolution through VJ and VDJ random recombination, as well as affinity maturation naturally occurring in vivo through somatic hypermutation. Until now, there are no in vitro or in-silico assays can precisely analyze the immunogenicity of antibody. In vivo assessments are usually used to evaluate the immunogenicity, of which the result will ameliorate design and engineering of antibody therapeutics to reduce the potential for inducing anti-drug antibodies.

### Generation of human antibodies by phage display

#### Overview of antibody phage libraries

Phage display is the first and still the most widely used technology for in vitro antibody selection. The strategy was developed based on the excellent work of George P. Smith in 1985 [14], who used recombinant DNA techniques to fuse foreign peptides with a coat protein (pIII) of bacteriophage M13 in order to display peptides on the bacteriophage surface. He then created “antibody-selectable phage vectors” and described an in vitro method that enabled affinity selection of antigen-specific phage-displayed antibodies from 10^8^-fold excess phage pools [92]. It was later discovered that scFv, small antibody formats, can be expressed on phage filaments. At the time, there were three different research institutions independently establishing phage-displayed scFv or Fab antibody libraries: the MRC Laboratory of Molecular Biology in the UK [13, 93, 94], the German Cancer Research Center in Germany [95], and Scripps Research Institute in the USA [96]. Since then, these phage-displayed antibody libraries have proven to be a reliable discovery platform for the identification of potent, fully human mAbs [97].

**Fig. 4:**
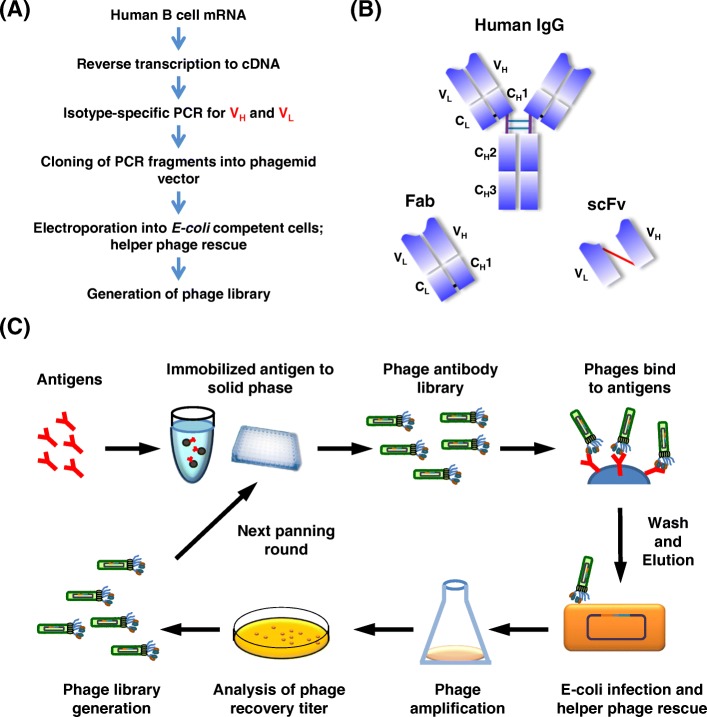
Construction and affinity selection with phage-display antibody library. **a** Outline of the procedure for constructing a phage-displayed antibody (Fab or scFv) library. **b** Structure of IgG molecule. Fab consists of the light chain and the first two domains of the heavy chain. scFv is composed of the variable heavy (V_H_) and variable light (V_L_) domains joined by a short flexible polypeptide linker. **c** Biopanning with a phage-displayed library. Initial pools of antibodies on the surface of phages are applied to antigens immobilized on a solid surface, e.g., ELISA plates or magnetic beads. Non-specific phages are removed by stringent washing. Antigen-bound phages are eluted and re-infected into *E. coli* to produce a subset of phages for the next cycle of panning. After several rounds, the antigen-binding clones are sufficiently enriched and individual clones can be selected for further analysis

The process of identifying mAbs from a phage-displayed library begins with antibody-library construction (Fig. 4a). The variable heavy (V_H_) and variable light (V_L_) polymerase chain reaction (PCR) products, representing the Ig gene-encoding repertoire, are ligated into a phage display vector (phagemid). High quality mRNA from human peripheral blood mononuclear cells (PBMCs) is reverse-transcribed into cDNA. The different V_H_ and V_L_ chain-region gene families are then amplified using specific primers to amplify all transcribed variable regions within the Ig repertoire [98, 99]. The format of antibodies in a phage-displayed library can be either scFv or Fab fragments (Fig. 4b); scFvs are composed of the V_H_ and V_L_ domain connected by a short flexible linker. Antibody Fab fragments displayed on the phage coat protein have comparably higher structural stability and can be readily converted to intact IgG antibodies, usually without impairing binding activity [100, 101]. The elegance of phage-displayed libraries is apparent in the linkage between antibody phenotype (specificity and sensitivity) and genotype (genetic information) via the phage particle. Due to the small size and high solubility (10^13^ particles/ml) of phage particles, repertoire sizes up to 10^11^ independent clones can be efficiently produced and displayed in a single library [102–104].

Gene repertoires for phage display libraries can be obtained from naïve or immunized animals, or the libraries may be synthetically constructed using randomized CDR sequences within fixed frameworks. Phage display naïve antibody libraries are constructed from rearranged V genes of IgM repertoires. Because the gene sequences are derived from B cells of human donors, the naïve libraries are relatively close to the human antibody germ line and have a low risk of immunogenicity. The main advantage of an immunized library over a naïve library is that antibody genes in the immunized library have undergone natural affinity maturation in vivo, allowing the development of high-affinity antibodies against the target. However, this approach requires that immunogenic response can be successfully induced by the antigen of interest, and new libraries must be prepared for each new target. Single large naïve [94, 104, 105] and synthetic [102, 106, 107] libraries have yielded high affinity antibodies (sub-nanomolar range) against a wide spectrum of targets. Therefore, such non-immunized libraries have the distinct advantages of avoiding issues with immunological tolerance in immunized mice, and they do not require new immunized libraries for each new target.

Currently, almost all widely accessible commercial libraries are based on highly diverse non-immunized gene repertoires, which allow selection of antibodies against a virtually unlimited number of targets [108]. It is worth noting that most antibody drugs that have undergone evaluation in clinical trials originated from a few company-owned libraries. These libraries include: Cambridge Antibody Technology’s (now MedImmune, a subsidiary of AstraZeneca) scFv-fragment library, Dyax Corp’s (now Shire) human Fab-fragment libraries, scFv and Fab libraries from XOMA, and the fully synthetic human combinatorial antibody scFv (HuCAL) and Fab (HuCALGold) libraries developed by MorphoSys [97].

#### Affinity selection of human antibodies

Antibody libraries are typically screened by iterative selection cycles to enrich target-binding phages, followed by amplification of the bound phages in *E. coli* cells. The affinity screening process for antibody libraries is called biopanning (Fig. 4c). Repeated rounds of selection allow for the enrichment of very rare antigen-binding phage clones, eventually resulting in the selection of the most highly specific binders. This stringent process is a critical feature of phage display that allows mAbs to be isolated in a period as short as a couple of weeks, far more quickly than the traditional hybridoma method [99, 109]. For in vitro selection, it is necessary to immobilize target antigens on a solid surface. Polystyrene surfaces with high protein binding capacity, such as 96-well immuno-plates and immuno-tubes, are widely used for antigen immobilization. Additionally, magnetic beads with protein G/A, streptavidin, maleimide or N-hydroxysuccinimide can be used to immobilize antigens and perform biopanning in solution.

The biopanning method is not only restricted to known recombinant proteins. In fact, the phage display technique may also be utilized to select antibodies against whole cells, unveiling previously unknown antigens on the tumor cell surface [110]. Cancer is an extremely heterogeneous disease, and only a few tumor cells with stem-like properties are able to initiate and sustain tumor development; these cells are often referred as tumor initiating cells or cancer stem cells (CSCs) [111]. Phage display technology is well suited for applications in CSC research, and several antibodies have already been identified from phage display libraries for their ability to bind known CSC markers, such as CD133 and CD44 [112, 113]. Moreover, novel CSC surface markers may be identified by selecting phage-displayed antibodies that bind to a CSC-like population and then identifying the corresponding target antigens [114, 115]. The use of tumor biopsy tissue as a biological material allows researchers to probe the tumor microenvironment, which may be highly relevant for clinical use. Phage display technology has been used to probe cancer tissue biopsies in order to generate antibody fragments that specifically recognize tumor subpopulations, such as CSCs and tumor-associated endothelial cells [116–118] as well as other clinically relevant tumor antigens [119].

Antibodies or antibody fragments have been referred to as targeted drug delivery “missiles” for their ability to direct homing of drugs to tumors [120]. For example, immunoliposomes have been demonstrated to provide conventional liposomal drugs with cancer targeting ability, which can increase the therapeutic efficacy of anticancer drugs [121]. Cellular internalization of the targeting ligand is an essential outcome for successful tumor-targeted liposomal drug delivery [99, 122]. For this reason, an efficient phage display-based selection approach was designed to map tumor internalizing epitopes, wherein a phage-displayed library was incubated with living cancer cells at 37 °C [123]. This method was successfully applied to rapidly identify several scFvs with high rates of internalization in several types of tumors; the target antigens were subsequently identified, and intracellular drug delivery systems were further developed [99, 124].

The identification of mAbs with phage display is an entirely in vitro process. Thus, it is not restricted by immunological tolerance, allowing for the identification of antibodies against poorly immunogenic antigens or those that are difficult to obtain using animal immunization methods (e.g., glycans or toxic agents). The in vitro nature of the assay can be especially useful when identifying specific antibodies against novel or gene-mutated pathogens in an outbreak of emergent infectious diseases [125–127]. The antigens on pathogens usually induce a strong immune response in patients, making it common for infected individuals to naturally produce high-affinity antibodies [128]. To obtain these antibodies, mRNA from the PBMCs of pathogen-infected people can be quickly collected and used as a gene repertoire for a phage-displayed library [129]. Such a library can allow for the rapid identification of high-affinity antibodies that may then be used as guides for vaccine design, or to develop therapeutic drugs and diagnostic reagents.

Moreover, the biopanning approach has been modified to isolate and identify a few antibodies with broad neutralizing activity against pandemic influenza virus [130, 131]. Chen et al. reported the establishment of a phage-displayed Fab library derived from the PBMCs of convalescent patients infected with a novel influenza A virus H7N9, which broke out in 2013. Using this library, antibodies targeting purified H7N9 virions were isolated [132]. Two human antibodies were found to exhibit high neutralizing activity against live H7N9 virus due to their interactions with the receptor-binding site of viral hemagglutinin antigens [132, 133].

The newly emergent Middle East respiratory syndrome coronavirus (MERS-CoV) induces a severe acute respiratory syndrome-like disease with an approximately 43% mortality rate [134]. To date, no vaccines or antiviral medications are available for the prevention or clinical treatment of MERS. A large phage-displayed human naive scFv library (Mehta I/II) with 2.7 × 10^10^ clones from the Dana-Farber Cancer Institute was used as a resource for the isolation of human antibodies against MERS-CoV [135]. In another project, a research group in Malaysia improved panning strategies with a naïve human scFv library (library size of 10^9^) to successfully identify mAbs specific to the MERS-CoV nucleoprotein [136].

Studies such as those described offer insights into the human antibody response to viral pathogen infection and provide examples of how the outbreak scene may be utilized to develop human antibody-based immunotherapies for the prevention and early treatment of viral pathogens [137].

#### The successful development of antibody drugs from phage display

Fully human therapeutic antibodies in current clinical use were discovered from either phage display or transgenic mice approaches [138]. Phage display has the advantage of allowing researchers to tailor critical characteristics of successful antibody drugs (e.g., affinity, specificity, cross-reactivity and stability). There are nine phage display-derived human antibodies currently approved by the US FDA for the treatment of human disease (Table 1), demonstrating the reliability of this technique as a platform for antibody discovery.

Adalimumab (Humira) was developed by BASF Bioresearch Corporation and Cambridge Antibody Technology. It was not only the first phage display-derived antibody granted a marketing approval, but adalimubab was also the first approved (2002) fully human mAb drug [139]. Adalimumab binds and suppresses TNFα and is approved to treat inflammatory diseases, such as rheumatoid and psoriatic arthritis, Crohn’s disease, and psoriasis. Adalimumab is the world’s best-selling drug [140] with sales of $19.9 billion in 2018 reported by AbbVie (Table 2). Cambridge Antibody Technology also identified human antibodies targeting BLYS (B lymphocyte stimulator) from phage display [141]. BLYS, a member of the tumor necrosis factor superfamily of cytokines, induces B cell proliferation and differentiation that positively correlate with systemic lupus erythematosus (SLE). This anti-BLYS antibody was named belimumab and marketed as Benlysta by GlaxoSmithKline, becoming the first drug approved (2011) for the treatment of SLE [142]. Founded in 1989, Cambridge Antibody Technology was acquired by AstraZeneca for $1.32 billion in 2006 [143].

Tyrosine kinase receptors, including EGFR and vascular endothelial growth factor receptor 2 (VEGFR2), play crucial roles in tumorigenesis, with higher expression and activation in tumors than in normal tissues. These characteristics make the receptors potentially valuable targets for drug development. Necitumumab (Portrazza) is an anti-EGFR human antibody that was identified by screening high EGFR-expressing epidermal carcinoma cells (A431) with a non-immunized phage Fab library of 3.7 × 10^10^ clones [105]. Necitumumab was approved in 2015 and is now a first-line therapy in combination with gemcitabine and cisplatin for the treatment of squamous NSCLC [144]. VEGFR2 is not only highly expressed in tumor endothelial cells, where it regulates tumor angiogenesis, but it also expressed on the surface of cancer cells. The anti-VEGFR2 human antibody, ramucirumab (Cyramza), was approved for the treatment of gastric cancer, metastatic NSCLC and metastatic colorectal cancer [145, 146]. The development of ramucirumab was initiated by using a phage-displayed human naïve Fab library (Dyax) containing 3.7 × 10^10^ independent clones for biopanning against the extracellular domain of human VEGFR2 protein [105]. Three Fab clones, D2C6, D2H2, and D1H4, were selected based on their specific binding to VEGFR2 with nanomolar affinity and their ability to neutralize VEGF-A-activated VEGFR2 signaling. Interestingly, these three Fab clones do not cross-react with murine VEGFR2 and share an identical V_H_ sequence [147]. After affinity maturation with stringent biopanning rounds, Fab clone 1121 (IMC-1121B) was selected and showed more than the 30-fold improvement of VEGFR2-binding activity. This clone was subsequently engineered into the human intact IgG_1_ version (ramucirumab), which has an affinity of 50 pM [148].

PD-L1, a cell-surface protein, binds to its receptor PD-1 on immune cells, downregulating T cell inflammatory activity to promote self-tolerance by the immune system. Many types of tumors have been found to express PD-L1 on the surface of cancer cells, using the immune-suppressing action to evade immune attacks. Avelumab (Bavencio) is fully human IgG_1_ lamda antibody against PD-L1, which was derived from a phage-displayed naïve Fab library (Dyax) [149]. Avelumab not only blocks PD-L1 binding to PD-1, but it also induces ADCC in cancers [150]. The later function differs from other immune checkpoint-blocking antibodies. The US FDA approved avelumab in 2017 for the treatment of urothelial carcinoma and Merkel-cell carcinoma, an aggressive type of skin cancer [151].

Psoriasis is a chronic autoimmune inflammatory disorder that causes skin cell overproduction and is characterized by raised, inflamed, red lesions and plaques that are accompanied by physical pain and itching. Guselkumab (Tremfya) is a fully human antibody developed by Janssen that neutralizes anti-IL-23. The HuCAL antibody library was used to generate guselkumab under a license from MorphoSys [152, 153]. In 2017, guselkumab was granted marketing approval by the US FDA for the treatment of plaque psoriasis [154].

Hereditary angioedema is a rare disease that results in spontaneous, recurrent, and potentially life-threatening attacks of swelling in various parts of the body [155]. The disease is commonly associated with deficiency or dysfunction of C1-esterase-inhibitor and with excessive bradykinin production caused by overactive plasma kallikrein [156]. Lanadelumab (Takhzyro) is a fully human mAb derived from the Dyax phage library; it directly binds the active site of plasma kallikrein to inhibit bradykinin production. Lanadelumab was approved in 2018 in the USA and Canada for prophylaxis against attacks of hereditary angioedema in patients aged ≥ 12 years [157].

The success of a drug development effort is highly dependent on obtaining patent protection for products and technologies while avoiding infringement on patents issued to others. Therefore, intellectual property rights for phage-display antibody discovery platforms comprise a changing landscape that greatly affects drug development. Currently (2019), almost all of the key patents regarding phage display technologies have expired, including the Breitling/Dübel (EP0440147) and McCafferty/Winter (EP0774511, EP0589877) patents that expired in 2011 in Europe [149]. The US patents covering Dyax and Cambridge Antibody Technology phage antibody libraries have also reached the end of their 20-year protection period (Table 3). The expiration of these patents will allow more companies to create phage display human antibody libraries, advancing the march of therapeutic antibodies into the clinic. The lifting of intellectual property constraints will also spur academic institutions to translate developed phage-displayed antibodies into the clinic.

**Table 3:** Key patents covering phage-displayed antibody libraries

Patent	Title	Company	Date filed	Date granted	Date of expiry
US5223409	Directed evolution of novel binding proteins	Dyax	03/01/1991	06/29/1993	6/29/2010
US5885793	Production of anti-self antibodies from antibody segment repertoires and displayed on phage	Cambridge Antibody Technology (CAT, now Medimmune)	12/02/1992	03/23/1999	3/23/2016
US6582915	Production of anti-self bodies from antibody segment repertories and displayed on phage	Cambridge Antibody Technology (CAT, now Medimmune)	11/28/2000	06/24/2003	09/23/2012
US5969108	Methods for producing members of specific binding pairs	Cambridge Antibody Technology (CAT, now Medimmune)	07/10/1991	10/19/1999	10/19/2016
US6172197	Methods for producing members of specific binding pairs	Cambridge Antibody Technology (CAT, now Medimmune)	06/07/1995	01/09/2001	01/09/2018
US5821047	Monovalent phage display	Genentech	6/5/1995	10/13/1998	10/13/2015
US6706484	Protein/(poly) peptide libraries (HuCAL libraries)	Morphosys AG	01/24/2000	03/16/2004	08/19/2016
US6753136	Methods for displaying (poly) peptides/proteins on bacteriophage particles via disulfide bonds (HuCAL GOLD libraries)	Morphosys AG	03/15/2001	03/21/2002	02/06/2021

Many researchers have begun to take advantage of these free technologies. For example, Wayne Maraso and colleagues at the Dana-Farber Cancer Institute in Harvard University have constructed two phage display libraries containing 12 billion (Mehta I) and 15 billion (Mehta II) human naïve scFv antibody phages. These libraries have been used to identify numerous human scFv antibodies against a variety of targets [158, 159]. James Marks’ group has also established a phage-displayed human scFv library containing 6.7 billion members at the University of California, San Francisco [98]. This library has yielded a panel of specific antibodies for membrane proteins and living tissues with sub-nanomolar affinity [98, 160, 161]. We have also established a phage-displayed human naive scFv library at the Institute of Cellular and Organismic Biology (ICOB) in Academia Sinica in Taiwan. The antibody gene repertoires of the ICOB phage antibody library were isolated from the PBMCs of 50 healthy human donors, producing a library size of 60 billion individual scFv clones. This collection has been successfully used to select antibodies that bind a wide spectrum of target antigens, including pure recombinant proteins, glycans, cancer cells and virus particles [99, 103, 104].

### Human antibody-producing mice

Transgenic animals provide a reliable platform for antibody drug development. Compared with other technologies for human antibody production, transgenic animals have several advantages, i.e., no need for humanization, more diversity, in vivo affinity maturation and clonal selection for antibody optimization. However, the large size of human Ig loci was a challenge during the development of transgenic mouse antibody technology. Additionally, the production of repertoires in transgenic mice that are similar or comparable those in humans requires diverse rearrangements combined with high expression of human V, D, and J segments [162]. To overcome these major challenges, different strategies have been successfully used to generate animals expressing human antibody repertoires (Table 4) [35, 36, 165].

**Table 4:**
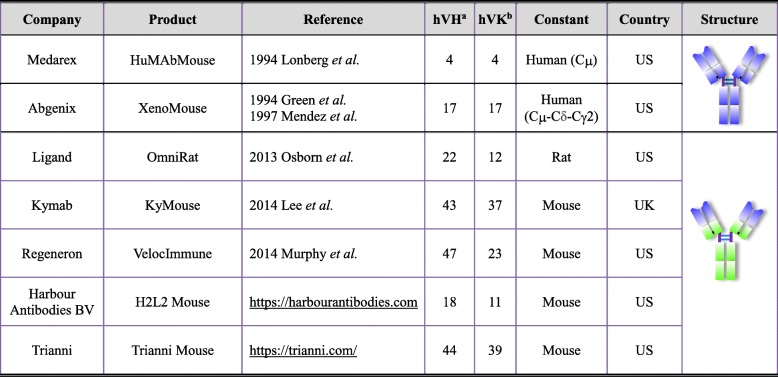
The major platforms of human antibody transgenic animals in the world [35–37, 163–165]

Blue: human sequence, Green: mouse sequence

^a^The number of human heavy chain variable region

^b^The number of human kappa chain variable region

#### Fully human antibody mice

The idea of producing human antibodies in transgenic mice was first suggested in 1985 when Alt et al. [166] proposed introducing human antibody genes into the mouse germline. This idea was unprecedented and provided a new direction for the development of human antibody production. In 1989, Brüggemann et al. [167] cloned the first human heavy chain construct, containing two human V_H_ genes, for diversity segments (D) linked to the human heavy chain joining cluster (J_H_), and the μ constant region. The 25 kb construct was micro-injected as a mililocus plasmid into fertilized murine eggs, allowing its random insertion into the murine genome. About 4% of B lymphocytes expressed human μ chain at detectable levels in these transgenic mice. In addition, hybridomas of human IgM antibody could also be established using this transgenic strain. In 1992, Taylor et al. cloned the human κ light chain [168] construct, containing one human kappa light chain variable (Vκ) gene, the human kappa light chain joining cluster (Jκ) and kappa constant region (Cκ). While the mice expressed the human heavy chain (V_H_-D-J_H_-Cμ-Cγ_1_) and the human kappa light chain, the amount of human antibody was less than 10% of total antibodies, so the expression of human antibodies was not compatible with expression of mouse endogenous Ig [168].

At a similar time, various murine Ig knockout mouse strains were generated. In 1993, Chen et al. knocked out murine J_H_ and Jκ genes with gene targeted deletion, inactivating mouse Ig [169, 170]. The human IgH and IgL transgenic mice were then crossed with murine IgH and IgL knockout mice in an attempt to create lines that could generate more diverse human antibodies. In 1994, the first human Ig -transgenic mice strain, HuMabMouse [35], was generated by Longberg et al. In this line, human IgH and IgΚ are expressed in murine IgH and IgΚ deficient mice. The entire human IgH genome is about 1.29 Mb and IgΚ is about 1.39 Mb, but the human Ig genome introduced into mice was less than 80 kb [35]. Since antibody diversity comes from germline V(D) J genes, it is reasonable that the introduction of more human variable genes will lead to more diversity of generated antibodies.

Likewise, Davies et al. [171] and Choi et al. [172] used yeast artificial chromosome (YAC) vectors in 1993 to respectively construct human IgΚ (~ 300 kb) and IgH (~ 85 kb) genes via yeast homologous recombination. In 1994, Green et al. [37] constructed human IgΚ (~ 170 kb) and IgH (~ 220 kb) genome YACs and successfully introduced them into mouse embryonic stem cells by yeast spheroplast-ES cell fusion. Furthermore, in 1997, Mendez et al. [36] introduced a larger human IgΚ (~ 700 Kb) or IgH (~ 1 Mb) YAC into mouse ES cells, crossing the human Ig mice with murine IgH and IgL knockout mice to generate XenoMouse [131]. As expected, the XenoMouse only express human antibodies rather than mouse antibodies [173]. While the development of this line has eliminated the interference from mouse endogenous Ig and expanded the human Ig genes, the efficiency of human antibody generation, Ig class-switching and somatic hypermutation still remain low, due to a lack of mouse constant region gene expression [158].

#### Chimeric human antibody mice

In order to overcome the drawbacks of a fully human heavy chain antibody, it is necessary to retain the original murine constant region. If a chimeric antibody with human Fab and murine Fc can be generated in mouse, the murine Fc would modulate the signaling for somatic hypermutation during antibody affinity maturation [158, 174, 175] and effector function of antibodies [174, 176]. Along these lines, Osborn et al. [163] linked human V_H_, D, and J_H_ genes to the rat constant region locus in 2013. The large segments of human IgH and IgL were subcloned and linked by bacterial artificial chromosome (BAC) and YAC techniques, followed by micro-injection of the minilocus plasmid into fertilized rat oocytes. Meanwhile, endogenous rat Ig loci were silenced with a zinc finger nuclease. The resulting rat strain (OmniRat) chimeric human exhibits antibody production, antigen affinity and somatic mutations similar to wild-type rats. In 2014, Lee et al. [164] utilized BACs with Cre/loxP recombination in mouse ES cells to generate a mouse strain with human V_H_-D-J_H_ and Vκ-Jκ inserted directly upstream of murine Cμ and Cκ regions (KyMouse). After antigen immunization, the KyMouse can process somatic hypermutation and produce high-affinity chimeric human antibodies. In another effort, Murphy et al. [165] used BACs to assemble large human Ig genes and serially micro-injected the constructs into mouse ES cells. The human IgH and IgL genes targeted and replaced the murine IgH and IgL, upstream of the constant region (VelocImmune mouse).

#### Development of successful antibody drugs from human antibody mice

Generation of human antibodies by transgenic animals has been accomplished by seven companies: Abgenix, XenoMouse (purchased by Amgen in 2005); Medarex, HuMAbMouse (purchased by Bristol Myers Squibb in 2009); Ligand, OmniRat; Kymab, KyMouse; Regeneron VelocImmune mouse; and the more recent Harbour Antibodies, H2L2 Mouse and Trianni Inc., Trianni Mouse [162, 165] (Table 4). The first antibody drug derived from transgenic mice was approved by the US FDA in 2006 [177], and as of 2019, 19 transgenic animal-derived antibody drugs [17–34, 177] generated by Xenomouse, HuMabMouse, and VelocImmune mouse are on the market (Table 5). Eight of the drugs are used for cancer treatment, while the others are for autoimmune or inflammatory diseases.

**Table 5:** US FDA-approved human mAbs

No.	Antibody	Brandname	Company	Approval^#^	Target	References	Technology
1	Adalimumab	Humira	Abbott_Laboratories	2002	TNFα	den Broeder et al. [178]	Phage display
2	Panitumumab	Vectibix	Amgen	2006	EGFR	Tyagi et al. [179]	XenoMouse
3	Ustekinumab	Stelara	Johnson & Johnson	2009	IL-12	Bartlett et al. [17]	HuMabMouse
4	Canakinumab	Ilaris	Novartis	2009	IL-1β	Church et al. [18]	HuMabMouse
5	Golimumab	Simponi	Johnson & Johnson/Merck	2009	TNFα	Zhou et al. [19]	HuMabMouse
6	Ofatumumab	Arzerra	GlaxoSmithKline (Genmab)	2009	CD20	Coiffier et al. [20]	HuMabMouse
7	Denosumab	Prolia, Xgeva	Amgen	2010	RANKL	Reddy et al. [21]	XenoMouse
8	Belimumab	Benlysta	GlaxoSmithKline	2011	BCAF	Ding et al. [180]	Phage display
9	Ipilimumab	Yervoy	Bristol-Myers Squibb	2011	CTLA-4	Morse et al. [22]	HuMabMouse
10	Ramucirumab	Cyramza	Eli Lilly (ImClone)	2014	VEGFR2	Krupitskaya et al. [181]	Phage display
11	Nivolumab	Opdivo	Bristol-Myers Squibb	2014	PD-1	Wolchok et al. [23]	HuMabMouse
12	Alirocumab	Praluent	Sanofi and Regeneron	2015	PCSK9	Roth et al. [24]	Veloclmmune Mouse
13	Daratumumab	Darzalex	Johnson & Johnson (Genmab)	2015	CD38	de Weers et al. [25]	HuMabMouse
14	Necitumumab	Portrazza	Eli Lilly (ImClone)	2015	EGFR	Kuenen et al. [182]	Phage display
15	Evolocumab	Repatha	Amgen	2015	PCSK9	Hirayama et al. [26]	XenoMouse
16	Secukinumab	Cosentyx	Novartis	2015	IL-17α	Chioato et al. [27]	XenoMouse
17	Olaratumab	Lartruvo	Eli Lilly	2016	PDGFRα	Chiorean et al. [28]	HuMabMouse
18	Atezolizumab	Tecentriq	Roche	2016	PD-L1	McDermott et al. [183]	Phage display
19	Avelumab	Bavencio	Pfizer	2017	PD-L1	Boyerinas et al. [184]	Phage display
20	Brodalumab	Siliq	Valeant Pharmaceuticals	2017	IL-17R	Papp et al. [29]	XenoMouse
21	Dupilumab	Dupixent	Sanofi and Regeneron	2017	IL-4R	Wenzel et al. [30]	Veloclmmune Mouse
22	Durvalumab	Imfinzi	Medimmune/AstraZeneca	2017	PD-L1	Antonia et al. [31]	XenoMouse
23	Guselkumab	Tremfya	Jassen Biotech	2017	IL-23	Sofen et al. [185]	Phage display
24	Sarilumab	Kevzara	Sanofi and Regeneron	2017	IL-6R	Huizinga et al. [32]	Veloclmmune Mouse
25	Erenumab	Aimovig	Novartis and Amgen	2018	CGRPR	Tepper et al. [33]	XenoMouse
26	Cemiplimab	Libtayo	Regeneron	2018	PD-1	Migden et al. [34]	Veloclmmune Mouse
27	Emapalumab	Gamifant	NovImmmune	2018	IFNγ	Al-Salama ZT [186]	Phage display
28	Moxetumomab pasudodox	Lumoxiti	MedImmune/AstraZeneca	2018	CD22	Kreitman et al. [187]	Phage display

^#^Year of the first US FDA approval

There have been seven US FDA-approved antibody drugs generated from the XenoMouse (Table 5). In 2006, the first one, panitumumab (Vectibix, Amgen, human IgG2/kappa), was approved to treat the EGFR-expressing metastatic colorectal cancer with wild-type KRAS [188]. This mAb blocks the interaction of EGFR and its ligands, resulting in the inhibition of EGFR signaling and induction of cancer cell apoptosis. Two antibody drugs from the XenoMouse are used for autoimmune dermatologic diseases. One, secukinumab (Cosentyx, Novartis, human IgG1), binds to proinflammatory cytokine IL-17α to reduce inflammation in psoriasis [189]. The other is brodalumab (Siliq, Valeant Pharmaceuticals, human IgG2), which binds to the IL-17 receptor to inhibit the action of IL-17 family cytokines. The two mAb drugs were approved by the US FDA for psoriasis treatment in 2015 and 2017, respectively.

From the HuMabMouse, there have also been eight antibody drugs approved by the US FDA (Table 5). Two drugs, ipilimumab (Yervoy, Bristol-Myers Squibb, human IgG1) and nivolumab (Opdivo, Bristol-Myers Squibb, human IgG4/kappa), are used for melanoma treatment; the drugs were approved in 2011 and 2014, respectively. Ipilimumab binds to CTLA-4, an immune checkpoint inhibitor, blocking its interaction with B7 on APCs and causing cytotoxic T lymphocytes to kill cancer cells [190]. Nivolumab recognizes to PD-1, reducing inhibitory signaling to rehabilitate the immune response of tumor-specific T cells in patients [191]. Notably, nivolumab was also approved for non-small cell lung cancer treatment in 2018. Among the mAb drugs derived from the HuMabMouse, some are used for autoimmune diseases. For example, ustekinumab (Stelara, Johnson & Johnson, human IgG1/kappa) binds to cytokines, especially the p40 subunits of IL-12 and IL-23, blocking proinflammatory signaling to ease inflammation. This drug was approved for severe plaque psoriasis [17] in 2009 and for Crohn’s disease [192] in 2016.

The VelocImmune mouse is a second generation transgenic chimeric mouse and has yielded four approved drugs (Table 5). Dupilumab (Dupixent, Sanofi and Regeneron, human IgG4) binds to IL-4 receptor and inhibits the IL-4 and IL-13 pathway, as an eczema treatment. Sarilumab (Kevzara, Sanofi and Regeneron, human IgG1) inhibits IL-6 signaling by binding to the IL-6 receptor (IL-6R), which otherwise would upregulate the release of rheumatoid arthritis-related factors from hepatocytes. The two drugs were both approved in 2017. Notably, despite having access to the XenoMouse and owning Cambridge Antibody Technology (the phage display company behind Humira), AstraZeneca paid over $120 million for a few breeding pairs of VelocImmune mice [193].

To improve the diversity of products and generate better antibody drugs, major development efforts have yielded models, such as the fully human antibody mouse and second-generation chimeric human antibody mice, over the 30 years since the first transgenic mouse was generated in 1989 [167]. The continued refinement and advancement of transgenic animals provides ever more possibilities for antibody drug development by global pharmaceutical factories.

### Single B cell antibody technology

In the human immune system, antibody responses are robust, highly specific, neutralizing and self-tolerant. Producing therapeutic human antibodies using the traditional hybridoma technique or transgenic mice requires long-term immunization procedures and screening, while the clinical use of murine antibodies may trigger severe immunogenic responses (such as HAMA) [194]. To avoid these obstacles, a technique for immortalizing human B cells with Epstein-Barr virus was developed [195–197]. This method is useful in certain situations, but it suffers from drawbacks, such as inefficiency in some patients and difficulties maintaining the stability of some transformed clones. While mice carrying human Ig genes have been created [35, 36, 163–165], the immune reactivity of these mice often cannot be triggered as robustly as natural human antibody responses. Thus, in emergent cases such as infectious diseases, single B cell antibody technologies have the major advantage of requiring only a few cells, allowing the highly efficient and rapid isolation of potential mAbs. Moreover, single B cell cloning preserves the biologically mediated heavy chain and light chain pairing, instead of the random pairing that is characteristic of mAbs from phage display antibody libraries. These randomly paired mAbs occasionally lose binding affinity or develop self-reactivity when transferred from scFv to intact IgG formats.

#### Identification and isolation of single B cells

Single B cells can be isolated from either PBMCs or lymphoid tissues using micromanipulation [198, 199], laser capture microdissection [200], and fluorescence-activated cell sorting [199, 201, 202]. Generally, mononuclear cells are purified from PBMCs or bone marrow by Ficoll-Paque density gradient centrifugation. Based on B cell expression of specific cell surface markers in different stages, isolation of single B cells by fluorescence-activated cell sorting is widely utilized, especially in the identification of rare and discrete B cell subpopulations. Antigen-coated magnetic beads [203] and fluorescence-conjugated antigens [204–206] are also often used to select antigen-specific B cells in a process known as antigen baiting. Neutralizing human mAbs against Puumala virus were generated from B cells isolated using antigen-coated magnetic beads [207]. Recently, antigen-conjugated fluorescent beads have been used to identify antigen-specific B cells [208]. Fluorescent virus-like particles of rotavirus served as antigen bait for single RV-specific B cells, which had been extracted from healthy rotavirus-infected infants or adult donors [209]. HIV envelope protein antigens have also been used to isolate antibodies that broadly neutralize HIV-1 [210, 211]. Moreover, isolation of dengue virus-specific memory B cells was reported [212, 213]. Thus, antigen baiting may be applied as a preliminary selection tool to be used on a polyclonal mixture.

#### Cloning of single B cells and screening of antibodies

After single B cell sorting, direct cloning of each Ig heavy chain and corresponding light chain should be performed [201, 202]. This step involves the use of nested or semi-nested reverse transcription-polymerase chain reaction (RT-PCR) for amplification of the variable heavy and light chain of each identified B cell. Usually, forward primers are directed toward IgH and IgL variable leader sequences and reverse primers are complementary to the Ig constant region [201, 214]. By optimizing different primer-set mixtures, the recoveries of the V_H_ and V_L_ may be improved [201, 215]. The genes are then cloned and expressed in mammalian cell lines for the immediate generation of recombinant mAbs. Following the detection of mAb reactivity, the characteristics of each generated mAb are determined. Furthermore, for high-throughput screening and evaluation of secreted mAbs with ideal reactivity, a cell-based microarray chip system [216] and microengraving techniques [217–220] have been described. The cell-based microarray chip system, immunospot array assay on a chip, enables the trapping of secreted antibodies by a chip that is coated with antibody against Ig, therefore, it is used to identify and recover specific antibody-secreting cells [216]. The microengraving method depends on the use of a soft lithographic technique to generate microarrays comprising the secreted antibodies of single cells [217]. These two approaches offer the advantages of early and rapid identification of clones with high affinity and specificity to the antigen of interest.

#### Generation of human antibodies by single B cell

In the face of threats from novel emergent pathogens, the rapid development of immunotherapies or insights into the diversity of antibody repertoire are beneficial, and single B cell sorting provides a highly efficient technology to achieve these goals. In the past, human mAbs have been generated by the single B cell method for bacterial, parasitic, virus infected or autoimmune diseases.

Among bacteria, *Bacillus anthracis* is one of the most concerning species. *B. anthracis* is a fatal pathogen that causes severe anthrax disease in humans and has been used as a biological weapon. Although antibiotics are available for anthrax treatment and as post-exposure prophylaxis, anti-anthrax protective antibodies from single human B cells will still be a crucial addition to the treatment toolkit [221, 222]. In an example targeting yeast infections, anti-*Candida* mAbs antibodies derived by the single human B cell method can enhance phagocytosis to protect against disseminated candidiasis [223].

The single B cell method has also successfully yielded anti-viral mAbs. Rapid isolation of dengue-neutralizing antibodies from human antigen-specific memory B-cell cultures [224] and characterization of antigen-specific B cells in the peripheral blood of DENV-immune individuals [213] were both reported. In another example, Iizuka and colleagues described the identification of cytomegalovirus pp65 antigen-specific human mAbs using single B cell-based antibody gene cloning [225]. For rotavirus, the single B cell method was performed to analyze the rotavirus antibody gene repertoire of VP6-specific B cells in naive and memory B cell subsets [226] and generate rotavirus-specific human mAbs by sorting single B cells from small-intestinal mucosa [227]. Human mAbs against zika virus NS1 have also been generated by the single B cell method [228]. Besides mAbs for bacterial and virus infection, the single human B cell method has also yielded a complement factor H (CFH) therapeutic antibody for cancer. The recombinant anti-CFH antibody can induce complement-mediated cytotoxicity (CDC) through complement activation and release of anaphylatoxins [229].

#### Development of single B cell-derived antibodies in clinical trials

Many virus-targeting mAbs are also currently in clinical trials. For example, Ebolavirus is a highly lethal pathogen that causes 25–90% mortality in humans. Therapeutic mAbs for ebolavirus infection have been derived from B cells of vaccinated human donors or survivors [230–232]. Impressively, human mAb114, which is derived from sorted memory B cells targeted to the Zaire ebolavirus glycoprotein, protects macaques when administered as late as 5 days after challenge [231]. Clinical trials for this drug are at Phase I (NCT03478891), Phase II and III (NCT03719586).

Acquired immunodeficiency syndrome is caused by HIV, and an estimated 36.9 million people worldwide are infected. HIV-1 envelope protein is an attractive therapeutic target for antibody and vaccine design. Five human mAbs against anti-HIV envelope protein have been generated by the single B cell approach and are under evaluation in clinical trials (3BNC117, Phase I/II; 10–1074, Phase I; VRC01, Phase I/II; PGT121, Phase I/II and N6, Phase I) [233]. A Phase I clinical trial (NCT02579083) is also being conducted on the prevention of sexual transmission of HIV-1 and herpes simplex virus by MB 66 combined with an anti-herpes simplex virus antibody (AC8) and an anti-HIV antibody (VRC01).

Different influenza viruses cause epidemics ever year, and influenza vaccines are the most useful measure to prevent seasonal influenza. Single B cell isolation for the generation of potent and broadly neutralizing anti-influenza antibodies has become a popular undertaking [234, 235]. MHAA4549A, a human mAb targeting the hemagglutinin stalk of influenza A virus was cloned from a single human plasmablast cell from an influenza virus vaccinated donor [236]. A Phase II clinical trial of MHAA4549A as a monotherapy for acute uncomplicated seasonal influenza A in otherwise healthy adults was recently completed (NCT02623322). CT P27, which contains two human mAbs (CT P22 and CT P23), was created by Celltrion and is in Phase II (NCT03511066). RG 6024, also named MHAB5553A, was generated by Genentech with a modified version of the single B cell isolation method [237]; it is currently under examination in Phase I (NCT02528903) trial. The Phase II trial for another antibody, TCN 032, was stopped in 2012 (NCT01719874).

Profiling of respiratory syncytial virus (RSV) antibody repertoires from the memory B cells of naturally infected adults [238] or generation of neutralizing antibodies from RSV-infected infants [239] has been undertaken as well. MEDI8897, an anti-RSV antibody developed by MedImmune, is currently being evaluated for safety and efficacy in Phase II clinical trial (NCT02878330).

Over the past decade, generation of mAbs by single B cell technology has become increasingly attractive. However, there are still no US FDA-approved therapeutic mAbs developed by this method that is used for clinical treatment of any disease. Although single B cell technology possesses several irreplaceable advantages, there are still challenges to be overcome. For example, the antigen labeling technique, the configuration of sorting antigens (e.g., monomer or dimer) and the design of primer sets are all important considerations for successful generation of mAbs. In the future, recovery of mAbs from single B cell platforms may be a powerful tool in combination with next generation sequencing for development of novel diagnostics, pharmacokinetic applications, and clinical therapeutics.

### Affinity maturation of antibodies

Antibodies identified from humanized, phage or transgenic methods are often further engineered, including the replacement of residues with binding liabilities in the binding region. In addition, point mutations in the antibody structure sometimes result in products with weaker antigen interactions than the original antibody (low affinity), but some mutations will result in stronger interactions (high affinity). The process of enhancing affinity for antigens is called affinity maturation. After V(D) J recombination, affinity maturation occurs in mature B cells with the help from helper T cells.

Affinity maturation is an important characteristic of the humoral immune response, which can result in antibodies with low picomolar affinity [240, 241]. High affinity is a crucial attribute of antibodies for the neutralization of cytokines or growth factor-induced signaling. Generally, for a mAb to be considered for therapeutic drug development, it should have an affinity of 1 nM or less for the target antigen [242]. Moreover, humanization of mouse mAbs frequently reduces affinity [243]. Thus, the use of affinity maturation is often a necessary step in antibody drug development [244].

#### Approaches for affinity maturation

Phage display and yeast display have been widely used for affinity maturation of antibodies, due to their amenability to easily screen for high affinity variants and to high throughput applications [81]. Methods for increasing antibody affinity can be broadly divided into two broad categories. The first is to generate a large randomly mutated library of CDR or entire variable domain sequences, followed by a selection of higher affinity variants from this large number of mutants. Another approach is to prepare small libraries by focused mutagenesis or hotspot mutagenesis that mimics in vivo affinity maturation. In this focused method, a high affinity variant is selected to be randomized at individual positions in each of the six CDRs or at a discrete point in the variable domain called the hotspot. The usual practice is to combine different mutations, resulting in small increases in affinity. The combination of these different mutations may have an additive or synergistic effect that can result in substantial increases in the affinity of the antibody to the antigen [245]. Phage display technology can be employed to identify high affinity antibodies in an antibody gene mutation library under stringent biopanning conditions, including decreased antigen amount, extended incubation and intensive washing steps or by competition with soluble antigen [104, 246]. Using phage display with V_H_ and V_L_ CDR3 mutation libraries, the affinity of anti-HER2 antibody was improved more than 1200-fold [97].

Diversification of antibody genes is the initial step of affinity maturation in vitro, and this step may be achieved using various strategies, such as random mutations, targeted mutations or chain shuffling [246]. Mutations can be randomly introduced into the variable regions of antibody genes by error-prone PCR in mutator *E. coli* strains [247, 248]. Chain shuffling approaches are those in which one of two chains, V_H_ and V_L_, is fixed and recombined with a repertoire of partner chains to produce a next-generation library [249]. Moreover, mutations can be introduced to particular regions of the antibody gene. This type of targeting mutation approach was employed to diversify CDR residues and was shown to be effective in ameliorating the affinity of antibodies [250]. Therefore, this method is more relevant to in vivo somatic mutations during B cell evolution because mutations accumulate more efficiently in the CDR than framework residues.

## Future perspectives

The field of therapeutic antibodies has undergone rapid growth in recent years, becoming a dominant force in the therapeutics market. However, there is still significant growth potential for the therapeutic antibody field. Traditionally, antibodies have been used for the treatment of cancer, autoimmune diseases, and infectious diseases. If the molecular mechanisms of a specific disease can be clearly elucidated and the specific proteins or molecules involved in pathogenesis can be identified, antibodies may provide an effective therapeutic option. For example, anti-CGRP receptor antibodies (erenumab, galcanezumab, or fremanezumab) have been developed for the prevention of migraine. Anti-proprotein convertase subtilisin/kexin type 9 (PCSK9) antibodies (evolocumab or alirocumab) are used for the treatment of hypercholesterolemia. Anti-fibroblast growth factor 23 (FGF23) antibody (burosumab) is used to treat X-linked hypophosphatemia. Anti-IL6R antibody (sarilumab and tocilizumab) can be used for the treatment of rheumatoid arthritis. Anti-Factor IXa/Xa antibody (emicizumab) is a valuable treatment for hemophilia A. Anti-von Willebrand factor antibody (caplacizumab) is approved for the treatment of thrombotic thrombocytopenic purpura, and other antibodies will be approved for new indications in the near future.

**Fig. 5:**
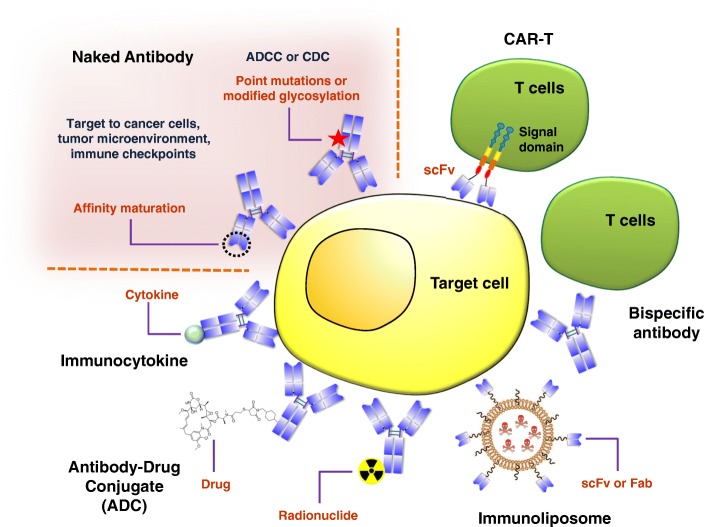
Schematic overview showing the development of antibody-based therapeutics for the treatment of cancer. Therapeutic antibodies can be roughly separated into two broad categories. The first category involves the direct use of the naked antibody for disease therapy. Antibodies in this category are used for cancer treatment and elicit cell death by different mechanisms, including ADCC/CDC, direct targeting of cancer cells to induce apoptosis, targeting the tumor microenvironment, or targeting immune checkpoints. For antibodies in the second category, additional engineering is performed to enhance their therapeutic efficacy. Some general approaches for the use of these antibodies include immunocytokine, antibody-drug conjugate (ADC), antibody-radionuclide conjugate (ARC), bispecific antibody, immunoliposome, and CAR-T

Therapeutic antibodies can roughly be separated into two broad categories (Fig. 5). In the first category, the naked antibody is directly used for disease therapy. Cancer treatments from this category may act through several different mechanisms, including mediated pathways (e.g., ADCC/CDC), direct targeting of cancer cells to induce apoptosis, targeting the tumor microenvironment, or targeting immune checkpoints. In mediated pathways, the antibody kills cancer cells by recruiting natural killer cells or other immune cells. Recently, new technological developments have been made to enhance the therapeutic effects of ADCC or CDC, such as antibody Fc point mutations [251–253] or modification of glycosylation [254–258] to improve cancer cell killing capabilities. The direct induction apoptosis in cancer cells has traditionally been the preferred mechanism for therapeutic antibodies. With regard to targeting the tumor microenvironment, antibodies can inhibit tumorigenesis by targeting factors involved in cancer cell growth. For example, Avastin targets vascular endothelial growth factor (VEGF) to inhibit blood vessel growth around the tumor, shutting down the supply of nutrients required for the cancer cell growth. Immune checkpoints have proven to be valuable targets for cancer treatment. In the future, studies evaluating synergistic effects of antibodies and chemotherapeutic drugs, radiotherapy or other biologic agents will greatly benefit the further development of antibody therapeutics. Furthermore, the identification of novel biomarkers may improve the efficacy and specificity of antibody-based therapy for human diseases.

In the second category of antibody drugs, additional modifications are made to the antibody in order to enhance its therapeutic value. Some general approaches include immunocytokines, antibody-drug conjugate, antibody-radionuclide conjugates, bispecific antibodies, immunoliposomes, and chimeric antigen receptor T cell (CAR-T) therapy. To create an immunocytokine, a selected cytokine is fused to an antibody to enhance delivery specificity [259]. Antibody drug conjugates consist of an antibody that targets a cancer-specific marker conjugated to the small molecule drug; the antibody enhances delivery to the tumor site, increasing the efficacy of the small molecule while reducing side effects by reducing non-specific toxicity to non-target tissues [260]. The antibody may also be conjugated to a radionuclide, in order to direct radiotherapy more specifically to the tumor site [261]. For bispecific antibodies, antibodies targeting two receptors are engineered to further enhance therapeutic effects [262]. Antibody-engaged effector cell functions may enhance the therapeutic efficacy of bispecific antibodies. With regard to immunoliposomes, the binding site of the antibody (scFv or Fab) is cleaved from the constant region and subsequently conjugated to different nano-drug delivery systems, such as liposomal drugs, to provide more specific targeting [263, 264]. Lastly, CAR-T involves inserting the gene for a chimeric T cell receptor-antibody targeting a specific cancer marker into T cells, such that the engineered cells target and kill cancer cells [265, 266]. In recent years, this approach has garnered major attention from the scientific and medical community due to its significant clinical benefits to cancer patients. In many cases, patients have experienced complete remission or even been completely cured of cancer [267–271].

Although new methods have been well-established for generating fully human antibodies, such as human antibody transgenic mice and human single B cell antibody techniques, phage display still has advantages as an antibody drug discovery platform, based on its efficient and economical in vitro selection methodology. Recently, some advanced techniques have been applied in antibody discovery, including high-throughput robotic screening [272], next generation sequencing [273] and single cell sequencing [274, 275]. These techniques are expected to greatly accelerate the identification of specific phage binders, facilitating mAb development for use in research, clinical diagnostics, and pharmaceuticals for the treatment of human disease.

By reviewing currently approved mAbs, one may easily see how sophisticated formats were developed in response to challenges posed by therapeutic indications. These mAb engineering solutions are highlighted by antibody-drug conjugates, glycoengineered mAbs, immunomodulators, bispecific mAbs, and CAR-T cells.

## Conclusions

Here, we summarize five technical platforms that are related to the production of therapeutic antibodies, including chimeric antibodies, humanization, phage display, transgenic mice, and single B cell antibody technology (Fig. 3d). Phage display, transgenic mice, and single B cell antibody technology have proven to be reliable methods for the generation of human antibodies. As enormous storehouses of antibody encoding genes (> 10^10^) with unknown properties, high quality (antibody diversity) phage antibody libraries are critical to the successful identification of therapeutic mAbs. In addition, an optimal selection from phage display libraries is dependent on target antigen quality, antigen immobilization, and tight control of binding and wash conditions. Furthermore, careful pre-screening design of conditions can tailor the characteristics of antibodies discovered from biopanning, including conformation specificity, epitope specificity, internalization, neutralization, and interspecies cross-reactivity. Currently, there are nine fully human antibodies that were discovered from phage libraries approved for therapy, and dozens more phage-derived antibody therapeutics are in clinical trials, waiting to enter the market [149] (Table 5).

In order to improve the quality of antibody drugs, researchers have developed several transgenic animals, including fully human mice and second-generation human chimeric mouse. The continued refinement and advancement of transgenic animals provide more options for antibody drug development in global pharmaceutical factories. All the transgenic-derived mAbs approved for therapeutic use have come from from three companies: Abgenix (XenoMouse) [36], Medarex (HuMAbMouse) [35], and Regeneron (VelocImmune) [165]. Depending on the immunization protocol, high affinity human antibodies can be obtained through selection of the clones generated in the animals. This selection is mainly accomplished by hybridoma technology. Currently, there are 19 approved human mAbs that were discovered from these three transgenic animals (Table 5).

Over the past decade, generation of mAbs from isolated single B cells has become an increasingly attractive approach. To date, no US FDA-approved therapeutic mAbs have been derived from this method; however, it possesses several major advantages, and ongoing challenges are currently being solved. The success of the method relies heavily on the antigen labeling technique, the configuration of sorting antigens (e.g., monomer or dimer) and the set of primers used for amplification. In the future, the recovery of mAbs from single B cells is expected to become a powerful tool in combination with next generation sequencing for diagnostics, pharmacokinetic application, and clinical therapeutics.

As a result of highly active development of antibody drugs in recent decades, mAbs have emerged among the major class of therapeutic agents for the treatment of many human diseases, especially cancers, immunological, infectious, neural and metabolic diseases. Sales growth and regulatory approval of mAb products were slow until the late 1990s when the first chimeric mAbs were approved (annual sales of $0.3 billion in 1997). With the subsequent approval of humanized and then fully human mAbs, the rate of product approvals and sales of mAb products has increased rapidly, with global sales revenue for all mAb products at $115.2 billion in 2018 (Fig. 1) [276]. The continued growth of mAb products in the coming years is expected to be a major driver of overall biopharmaceutical product sales.

## Data Availability

Not applicable.

## Abbreviations

ADCC: Antibody-dependent cellular cytotoxicity
BAC: Bacterial artificial chromosome
BLYS: B lymphocyte stimulator
CALCRL: Calcitonin receptor-like receptor
CAR-T: Chimeric antigen receptor T cell
CDC: Complement-mediated cytotoxicity
CDR: Complementary-determining region
CGRP: Calcitonin gene-related peptide
CLL: Chronic lymphocytic leukemia
CoV: Coronavirus
CSC: Cancer stem cell
CTLA-4: Cytotoxic T-lymphocyte–associated antigen 4
Cκ: Κappa constant region
D: Diversity segment
EGFR: Epidermal growth factor receptor
ES cell: Embryonic stem cell
Fab: Antigen-binding fragment
Fc: Fragment crystallizable region
HAMA: Human anti-mouse antibody
HER2: Human epidermal growth gactor receptor 2
HIV: Human immunodeficiency virus
ICOB: Institute of Cellular and Organismic Biology
Ig: Immunoglobulin
J_H_: Heavy chain joining cluster
Jκ: Kappa chain joining cluster
mAb: Monoclonal antibody
MERS: Middle East respiratory syndrome
MM: Multiple myeloma
MSI-H: Microsatellite instability high
NSCLC: Non-small cell lung cancer
PBMC: Peripheral blood mononuclear cell
PCR: Polymerase chain reaction
PD-1: Programmed cell death protein 1
PD-L: Programmed death-ligand
RAMP1: Receptor activity-modifying protein 1
RSV: Respiratory syncytial virus
RT-PCR: Reverse transcription-polymerase chain reaction
scFv: Single chain fragment variable
SLE: Systemic lupus erythematosus
TNFα: Tumor necrosis factor α
US FDA: United States Food and Drug Administration
VEGFR2: Vascular endothelial growth factor receptor 2
V_H_: Variable heavy chain
V_L_: Variable light chain
Vκ: Kappa variable light chain
YAC: Yeast artificial chromosome
